# DEEP picker is a deep neural network for accurate deconvolution of complex two-dimensional NMR spectra

**DOI:** 10.1038/s41467-021-25496-5

**Published:** 2021-09-01

**Authors:** Da-Wei Li, Alexandar L. Hansen, Chunhua Yuan, Lei Bruschweiler-Li, Rafael Brüschweiler

**Affiliations:** 1 Campus Chemical Instrument Center, The Ohio State University, Columbus, OH USA; 2 Department of Chemistry and Biochemistry, The Ohio State University, Columbus, OH USA; 3 Department of Biological Chemistry and Pharmacology, The Ohio State University, Columbus, OH USA

**Keywords:** Computational biophysics, Machine learning, Biophysical chemistry

## Abstract

The analysis of nuclear magnetic resonance (NMR) spectra for the comprehensive and unambiguous identification and characterization of peaks is a difficult, but critically important step in all NMR analyses of complex biological molecular systems. Here, we introduce DEEP Picker, a deep neural network (DNN)-based approach for peak picking and spectral deconvolution which semi-automates the analysis of two-dimensional NMR spectra. DEEP Picker includes 8 hidden convolutional layers and was trained on a large number of synthetic spectra of known composition with variable degrees of crowdedness. We show that our method is able to correctly identify overlapping peaks, including ones that are challenging for expert spectroscopists and existing computational methods alike. We demonstrate the utility of DEEP Picker on NMR spectra of folded and intrinsically disordered proteins as well as a complex metabolomics mixture, and show how it provides access to valuable NMR information. DEEP Picker should facilitate the semi-automation and standardization of protocols for better consistency and sharing of results within the scientific community.

## Abstract (web summary)

The analysis of NMR spectra of complex biochemical samples with respect to individual resonances is challenging but critically important. Here, the authors present a deep learning-based method that accelerates this process also for crowded NMR data that are non-trivial to analyze, even by expert NMR spectroscopists.

## Introduction

Multidimensional NMR spectroscopy is a powerful and versatile method for the quantitative characterization of a wide range of molecular systems ranging from small molecules to large biomacromolecules and their complexes^1,2^. A spectrum can consist of several hundred to thousands of cross-peaks manifested as localized multidimensional spectral features that in the case of 2D NMR belong to individual pairs of atoms that possess a nuclear spin. Identification and quantitative characterization of cross-peaks critically affect all downstream analyses and can have a major impact on data interpretation. Each cross-peak is characterized by the position of its center (i.e. frequency coordinates corresponding to chemical shifts), its peak shape along each dimension (usually Voigt shape with variable amounts of Lorentzian or Gaussian components), and its peak amplitude (or volume). The parameters that define the cross-peaks represent the chemical and biological information of interest about the molecule(s) present in the sample. The analysis of an NMR spectrum invariably involves some or all of the following steps: (i) identification of the complete set of cross-peaks, known as peak picking; (ii) assignment of each cross-peak to the atoms it belongs to; and (iii) quantification of each cross-peak by the determination of the peak amplitude or volume. Despite many years of progress, the above steps can only be partially automated. This applies in particular to spectra of large molecular systems or complex mixtures containing many cross-peaks that tend to overlap, which makes their spectral deconvolution challenging without expert human assistance. However, due to the large number of cross-peaks, such work can be tedious, time-consuming, and subjective with results differing between experts and labs, thereby limiting the transferability of the analysis within the research community. This makes the availability of an approach necessary that accomplishes the above tasks both with high accuracy and high reproducibility.

Different methods have been proposed for peak picking and spectral deconvolution. The simplest approach is to select local maxima as peak positions. However, because of spectral noise, not all local maxima belong to true peaks. Moreover, in crowded regions, some peaks may not correspond to maxima because of the close vicinity of larger peak(s) with which such shoulder peaks overlap. To address these formidable challenges, numerous approaches have been developed in the past. Early methods focused on criteria based on signal intensity, volume, signal-to-noise ratios, and peak symmetry^3–11^. Other peak picking methods exploit various forms of matrix factorization^12–14^, or singular value decomposition^15^. Another approach models spectra as multivariate Gaussian densities followed by filtering with respect to peak intensities and widths^16–18^. In these methods, certain spectral features are extracted after pre-processing and assessed following a set of rules to determine whether a data point is considered as a peak or not. Because these rules are defined based on experience, they often involve a lengthy trial and error process to decide which features best describe various types of peaks in different types of spectra. Moreover, each feature requires criteria that can depend on many parameters, especially when noise and other artifacts are present, which must be fine-tuned manually. Although these peak picking methods have shown steady improvements, much of the NMR community still relies at least in part on manual peak picking, whereby the final result is dependent on the expertise and judgment of the human NMR spectroscopist(s) working on the project. The latter currently still outperforms the best peak picker software.

Neural network-type machine learning methods introduced the concept of end-to-end learning where the machine is only given the input and the correct result. Thus, a machine-learning model is trained instead of hardcoded for the given data, where neural networks discover the underlying key features and automatically derive the most accurate set of empirical rules using a general-purpose algorithm. While a traditional neural network typically contains only one or two hidden layers^19,20^, deep neural networks (DNN)^19^ are composed of many simple but nonlinear layers that starting from the raw input each transform the previous layer’s representation into a new representation at a higher, more abstract level. With sufficient depth, deep learning can develop extraordinarily complex functions capable of discovering intrinsic structures in high-dimensional data that are invisible to the human eye. In the past decade, deep learning had an important impact on many different fields outperforming traditional algorithms or even humans, such as in image labeling, speech recognition, and natural language understanding^20^. Although NMR data possess many unique properties, machine learning has started to make in-roads^21^, such as for spectral reconstruction of non-uniformly sampled (NUS) datasets^22,23^, spectral denoising^24^, chemical shift prediction^25–28^, and also peak picking^29^.

Here, we introduce a sophisticated NMR spectral peak deconvolution method, based on a Deep Neural Network, called DEEP Picker (as an abbreviation for Deep nEural nEtwork Peak Picker). Its performance is demonstrated for different types of 2D NMR spectra of folded and intrinsically disordered proteins in solution and a mouse urine sample containing spectral regions with variable degrees of spectral overlaps.

## Results

### Generation of the training set

Since for every deep learning project the size and quality of database information are essential components, the exponential growth of data repositories over the past decade has been one of the major drivers for the rapid progress in deep learning. Such data can be either obtained from the real world (e.g. image libraries) or be synthetically generated entirely in silico, such as in data augmentation techniques^30^. A critical part of the successful training of a neural network is the availability of a large amount of high-quality training data that comprehensively mirror the envisioned applications. This includes an optimal class balance of the training data by ensuring that the distribution of training data among all classes is unbiased, otherwise, DNN accuracy for the identification of members of the underrepresented class(es) will be reduced^31,32^. As an example, for NMR peak picking, if the peaks in the training set have dominantly Gaussian lineshape, the resulting DNN is more likely to fail when applied to Lorentzian peaks and vice versa.

In order to ensure a large database of class-balanced, high-quality training data, we built our own database consisting of synthetic 1D NMR spectra with different peak widths, shapes, and peak overlaps. As discussed below, our 1D NMR-trained DNN can be deployed also to higher dimensional spectra. Because for synthetic spectra the parameters of the individual peaks are accurately defined, their use as a training set has the distinct advantage over an experimental training set that the “ground truth” is by definition known. A synthetic database has the additional advantage over an experimental database in that it allows almost unlimited coverage by sampling many more different peak shapes and overlap scenarios without requiring a human expert’s input for peak classification. The shapes of all synthesized NMR peaks generated here follow a Voigt profile^33^, which corresponds to the convolution of a Lorentzian and a Gaussian peak shape with the two shapes present in different amounts. The rationale for the Voigt profile is that in solution NMR the natural lineshapes are in good approximation Lorentzian, but after apodization using commonly used window functions, such as the 2*π*-Kaiser window function, the peaks acquire a Voigt profile with some Gaussian component with improved spectral resolution. The amount of the Lorentzian component in the final peak shape depends on the natural linewidth, i.e. 1/(*πT*_2_) where *T*_2_ is the transverse relaxation time. In our training set, the number of points per peak (PPP), which is given by the number of data points that sample the peak’s full-width at half-height (FWHH) (i.e. FWHH/(digital resolution)), is allowed to vary either from 6 to 20 points or from 4 to 12 points, whereby the former is typical for protein and the latter for metabolomics spectra. The Lorentzian component can vary from 0%, corresponding to a pure Gaussian lineshape, to 100%, corresponding to a pure Lorentzian lineshape. It is worth mentioning that for a given spectrum, both synthetic and experimental, PPP can be easily adjusted to meet the above criterion by adjusting the amount of zero-filling prior to Fourier transformation.

**Fig. 1:**
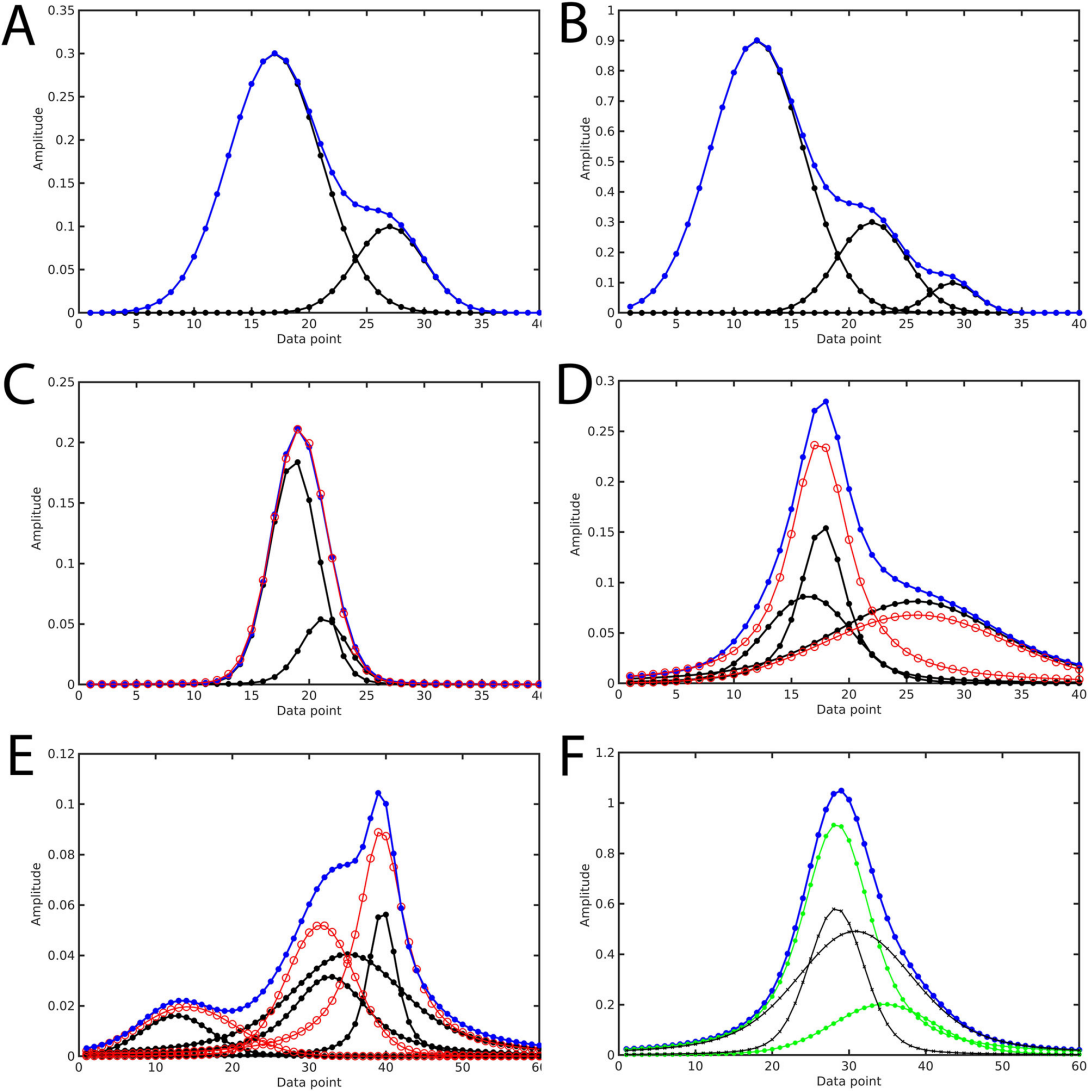
Examples of 1D NMR training sets of convoluted NMR spectra (blue) and their deconvolutions (black, red). **A** Sum spectrum (blue) that can be unambiguously deconvoluted into two individual overlapping peaks (black). **B** Sum spectrum (blue) that can be unambiguously deconvoluted into three individual overlapping peaks (black). **C** Sum spectrum (blue) of two overlapping peaks (black) can be accurately represented by a single peak (red). **D** Sum spectrum (blue) generated from three distinct peaks (black), but can also be accurately explained by only two peaks (red). **E** Sum spectrum (blue) generated from four distinct peaks (black), but can also be accurately explained by only three peaks (red). **F** Sum spectrum (blue) can be deconvoluted equally well into two distinct peak pairs (one with black crosses and one with green circles).

To generate synthetic 1D spectra for the purpose of the training and validation of the DEEP Picker, a database of peak pairs with random separation and amplitude was generated first. In this synthetic database, like in experimental spectra, a subset of peaks may overlap so strongly that they are impossible to distinguish, e.g. the spectrum resulting from two overlapped peaks might be impossible to uniquely deconvolute and, in the presence of some noise, can be equally well represented by a single peak. For the training of the neural network, it is important that a spectrum is assigned to deconvoluted peaks in a way that the neural network has a realistic chance to identify them, otherwise it is likely to fail in practice when encountering such types of ambiguous situations. Previous work^29^ mostly relied on peak lists generated by expert NMR spectroscopists to make this distinction, which implicitly makes human judgment an important part of the neural network training process. Our strategy is to use a well-defined mathematical criterion instead in the form of nonlinear peak fitting as the gold standard. Specifically, we apply nonlinear peak fitting using MATLAB assuming a single peak on all potential peak pairs. When the maximal difference in amplitude for all points of the original and the fitted peak is <3% of the peak amplitude, a synthetic peak pair is excluded from the training set. Figure 1A, B shows examples of two and three overlapped peaks that can be uniquely identified and Fig. 1C shows a case where the superposition of the two black peaks can be also explained by a single peak with minimal error. In experimental spectra, most peaks will not have a perfect Voigt shape because of noise, apodization, baseline distortion, and small phase errors, which may also cause asymmetric peak shapes. To prevent the trained neural network from picking only perfectly shaped Voigt-shaped peaks, we intentionally label certain strongly overlapped peak pairs as a single peak if the maximal absolute error is <2% (after peak fitting with a single peak) and the peak widths of the two peaks differ by less than a factor 1.5. Figure 1D shows an entry of the synthetic peak database for which the profile was generated from three peaks but assigned to only two distinct peaks (red peaks).

Next, we generated more complex synthetic spectra representing 3–5 peaks by randomly adding peaks and peak pairs from the original database. After this process, we performed nonlinear peak fitting to determine whether the generated spectrum can be explained (again within 3% maximal error) in a robust manner with a smaller number of peaks than the number used to generate it. If this was possible, the spectrum was removed from the database. Figure 1E shows an example of such a spectrum, which was added to the training set, generated from four overlapped peaks (black peaks) but assigned only to three well-defined distinct peaks (red peaks). These 1D spectra were then combined to form spectra with 300 data points with 3–9 peaks. Our final training and validation sets consist of 5000 and 500 of these 1D spectra, respectively.

Accurate identification of shoulder peaks is one of the most challenging tasks for any peak-picking algorithm. Unlike main peaks, shoulder peaks do often not belong to local maxima of the full spectrum, which makes their identification along with the accurate determination of their positions and amplitudes significantly harder. Figure 1F depicts the profile of two overlapped peaks along with two distinct peak deconvolutions, which both achieve <0.1% maximal error. The position of the main peak (left peak) is well defined, but the shoulder peak has a large positional uncertainty. Such situations need to be taken into account in the design of DEEP Picker as is discussed in the following section. We did not find it necessary to include spectral artifacts in our training sets. Certain potential artifacts are best addressed during spectral processing, such as baseline correction and apodization. Proper phase correction (0th and 1st order) is also important, although DEEP Picker can tolerate phase errors of a few degrees, which is realistically achievable in practical applications. Residual water signals and *t*_1_-noise can be identified before or after peak picking as discussed below.

### Design and training of DEEP Picker

Inspired by widely used image recognition and image labeling neural networks^34^, DEEP Picker runs a point-by-point prediction of the input spectrum on top of a sliding window using stacked convolutional layers^35^. DEEP Picker assesses every data point along the spectrum as either a peak or a non-peak and if assessed as a peak, it will also predict peak shape and amplitude. To boost the performance of the above algorithm for NMR spectral analysis, we made two changes. First, to accommodate the peak positional uncertainty, instead of labeling just the data point closest to the predicted peak position, in our training set we define the three closest points to the predicted peak position as “peak” and all other data points as “non-peak”. This permits accurate peak identification even when the predicted peak position is less well defined, e.g. in the presence of strong overlap. At the same time, the score for the successful peak prediction is increased. In this way, the neural network can be prioritized to predict other peaks accurately instead of trying to provide exact locations of peaks that have intrinsically elevated positional uncertainties. Second, the prediction of peak parameters of standalone peaks or overlapping peaks whose amplitudes substantially exceed those of their overlapping partner peaks can be accurately achieved, whereas peak parameter prediction of peaks, such as shoulder peaks, who are weaker than their overlapping neighbors is naturally much harder. We found that it is beneficial to predict the peak parameters of these two different types of peaks by using separate neural network components in the output regressor layer, which can be achieved by using different labels of the two types of peaks. This led to three different classes of output in our training and prediction framework for each point of a spectrum, namely “Class 2 peaks”, which are spectral features that can be explained by single peaks or peaks that dominate their overlapping neighbor peaks, “Class 1 peaks”, which are peaks that have overlap and are dominated by their overlapping neighbor peak(s) in terms of peak amplitude and volume and are usually manifested as shoulder peaks, and “Class 0 non-peaks”, which are spectral points that do *not* correspond to a peak center.

**Fig. 2:**
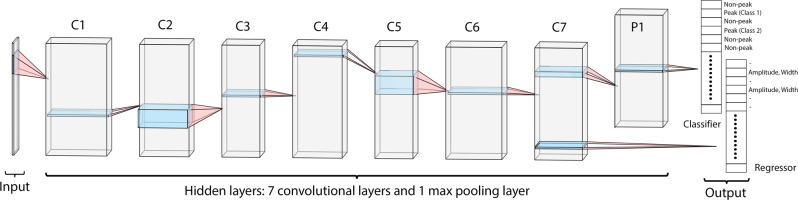
Architecture of the deep neural network peak picker (DEEP Picker), which is composed of seven 1D convolutional layers with rectified linear (ReLU) unit activation functions (C1–C7), one max-pooling layer (P1), one convolutional layer with a SoftMax activation function to classify every data point, and one convolutional layer with linear activation function to predict the peak position at the sub-pixel resolution, peak amplitude, peak width, and the Lorentzian fraction of its peak shape. The input of DEEP Picker is an *N* × 1 tensor (column vector), where *N* is the number of data points of the 1D input spectrum. Hidden layers and output layers all have the same dimension *N* as input. The depths of the 8 hidden layers (from C1 to C7, P1) are 40, 20, 10, 20, 10, 30, 18, and 18. Their kernel sizes are 11, 1, 11, 1, 1, 11, 1, and 3 and they are 1 for both the classifier and regressor. As is common in machine learning, all kernels are applied *N* times in a sliding window fashion with each layer having multiple kernels (note that in the figure for each layer only a single kernel is indicated for a given position). The output classifier layer (top right) yields the prediction of whether a data point is a Class 2 peak, Class 1 peak, or Class 0 non-peak. The output regressor layer (bottom right) yields the predicted peak parameters for all peaks (Class 2 and 1).

The neural network was implemented and trained using TensorFlow v1.3^36^ taking 1D spectra as input. The architecture of DEEP Picker is illustrated in Fig. 2. After hyper-parameter tuning, DEEP Picker contains 7 hidden convolutional layers, 1 hidden max-pooling layer, and two parallel output layers with a total of 8037 trainable parameters. In typical Convolutional Neural Networks for image classification and object detection, max pooling is combined with convolutional layers to achieve location invariance of features. By contrast, location invariance generally does not apply here, since a shift of one peak (or feature) will generally affect the interpretation of nearby peaks (or features). Therefore, we do not use max-pooling except for the penultimate layer. A convolutional layer with SoftMax activation^37^ called the output classifier layer, is utilized to classify every data point, which assigns an individual score for all three peak classes (2, 1, or 0), which are then normalized for each data point so that their sum is 1. The class with the maximal score is then chosen as the predicted class with the numerical score as a quantitative measure of confidence of the predicted class for each data point of the input spectrum. For any data point predicted to be a peak (Class 2 or 1), DEEP Picker will also predict the sub-pixel peak position relative to the on-grid points, peak amplitude, peak width, and the Lorentzian vs. Gaussian components to the Voigt shape using another convolutional layer, called output regressor layer. It is worth mentioning that all kernels were applied multiple times, i.e. across the full input spectrum in a sliding window fashion and each convolutional layer has multiple kernels although in Fig. 2 only one kernel operating at an arbitrarily chosen position is illustrated for each layer. The loss function is the mean squared error (MSE) for the regressor and cross-entropy for the classifier. The loss value (training target) is the weighted average of the cross-entropies of the three classes of data points and MSE of the two classes of peaks with the weights provided in Table S3. DEEP Picker was trained using the Adam optimizer with a learning rate of 0.002 for 4000 epochs^37^. All data were used simultaneously in a single batch. The performance of the validation set was monitored in this process to prevent potential overfitting. However, since the relatively small size of our neural network compared to the size of our training set, overfitting is not an issue. The output classifier layer (top-right) assigns to every spectral data point output in the form of either a Class 2 peak, Class 1 peak, or a Class 0 non-peak. The output regressor layer (bottom-right) predicts peak parameters (amplitude, linewidth, etc.) for any Class 2 or 1 peak. Because we label 3 data points to be a peak for each true peak in the training set, DEEP Picker will usually predict three consecutive data points as peak for well-defined peaks. However, for a peak with large positional uncertainty, such as a strongly overlapped peak, DEEP Picker might assign peaks to regions with fewer or more than three data points. In either case, the application of a non-maximum suppression algorithm^34,38^ for post-processing only keeps a (single) data point that has the highest score for each region as further explained below.

**Fig. 3:**
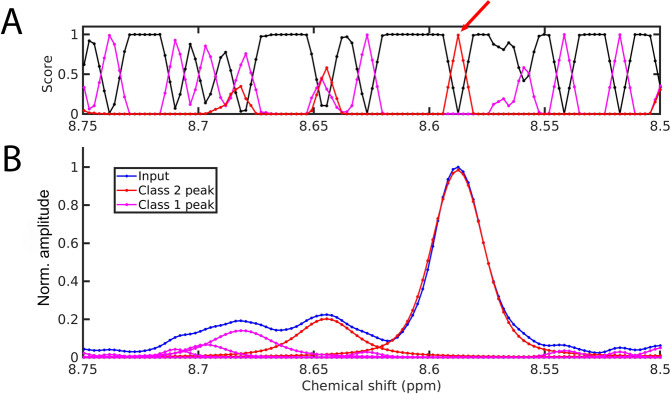
Peak prediction by DEEP Picker for K-Ras ^15^N–^1^H HSQC for part of a cross-section along the direct ^1^H dimension. **A** Prediction score of Class 2 peaks (red), Class 1 peaks (magenta), and Class 0 non-peaks (black) from the output classifier layer after a 3-point moving average. The class with the highest score is the class assigned to a given data point after non-maximal suppression (see text). **B** Input spectrum (blue) together with reconstructed individual Class 2 peaks (red) and Class 1 peaks (magenta).

The 1D DEEP Picker was then tested for a 1D ^1^H cross-section (along *ω*_2_) of an experimental 2D ^15^N–^1^H HSQC spectrum of K-Ras, which is a globular protein with 169 residues. Figure 3A shows the point-by-point prediction of the output classifier layer where the red, magenta and black lines are scores for the Class 2 peaks, Class 1 peaks, and Class 0 non-peaks, respectively. For each data point, the class with the highest score is taken as the predicted class. The sum of scores for Class 2 peaks and for Class 1 peaks is taken as a confidence level score of the picked peaks (see Fig. S8). This helps focus subsequent visual inspection on low-scoring peaks for their potential removal from further analysis. For example, Class 2 has the highest score for three consecutive data points around 8.59 ppm (indicated by a red arrow) and, hence, all three data points are predicted to be Class 2 peaks. Application of the non-maximum suppression algorithm will then suppress low-confidence predicted peaks that are direct neighbors of predicted peaks and only the middle data point with a score around 1.0 is kept since the scores of the two neighboring data points are only around 0.6. Once this middle data point has been identified as a peak, the deconvoluted peak is generated at sub-pixel position resolution along with its peak amplitude, peak width, and the fraction of Lorentzian vs. Gaussian components obtained from the output regressor layer. In Fig. 3B, red lines correspond to reconstructed individual Class 2 peaks from the prediction, including the peaks at 8.59 and 8.65 ppm. Magenta lines correspond to reconstructed individual Class 1 peaks (shoulder peaks) by the same method and the sum of the red and magenta spectra corresponds to the input spectrum.

Because DEEP Picker is a local feature-based predictor, it also assigns Class 2 or 1 peaks to noise features that are close to the baseline in regions without signal. Such noise peaks are subsequently removed if they are below a peak amplitude cutoff based on an automated global noise level estimator (see Supporting Information for details). Similarly, DEEP Picker may also predict a point with a small deviation from an otherwise smooth profile to be a separate peak. Because the predicted amplitude of such a peak will be small, it can be filtered out using the same type of peak amplitude cutoff.

### Generalization to 2D spectra

Because 2D NMR cross-peaks can have a much larger number of different peak shapes and overlap patterns than 1D spectra, the training set would need to be extremely large to achieve a robust neural network-based peak picker. Instead, we apply the DEEP Picker separately to all rows and columns of a 2D spectrum and combine the scores for 2D cross-peak identification. In order for a 2D data point to be identified as a cross-peak, the data point must be assigned by DEEP Picker to a 1D peak in both its cross-sections along *ω*_1_ (column) and *ω*_2_ (row) (exceptions will be discussed below). Peak width, sub-pixel position resolution, and percentage of Lorentzian vs. Gaussian components to the Voigt profile along the two dimensions are taken directly from the corresponding 1D prediction whereas the peak amplitude is obtained as the average of the two 1D predictions and the peak confidence level score is calculated as the lower of the two 1D confidence level scores.

**Fig. 4:**
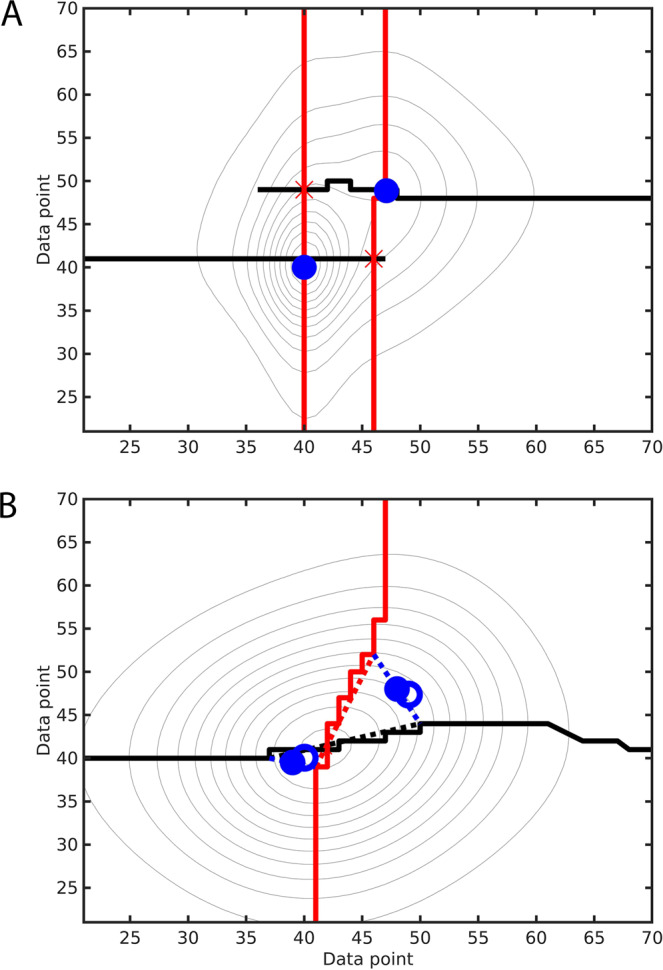
Contour plot of the synthetic 2D spectrum representing two overlapping cross-peaks (blue circles). First, DEEP Picker predicts 1D peak positions for each column (row) labeled in black (red). **A** Next, the 2D peak-picking algorithm uses intersections of black and red lines to define 2D cross-peaks, while removing false positive peaks (red crosses) using the approach described in the text. **B** If both black and red lines deviate from perfect vertical and horizontal lines, respectively, the 2D peak-picking algorithm will replace the intersection peak with two cross-peaks (blue filled circles) near the true positions (blue open circles) using the approach described in the text.

2D spectral peak picking is illustrated in Fig. 4 using a synthetic spectrum consisting of two overlapping cross-peaks where the true peak positions are indicated by blue circles. Figure 4A shows how the two cross-peaks at locations (40,40) and (48,48) are picked correctly. In addition, the row-based 1D peaks (bold black lines) identified by DEEP Picker also intersect with the column-based 1D peaks (bold red lines) at the locations (40,48) and (48,40), which would cause the prediction of these two additional cross-peaks (red crosses) that are however false. The 2D peak-picking algorithm is able to identify and remove these types of false-positive cross-peaks based on the fact that along both their rows and columns they behave as 1D shoulder peaks (Class 1 peaks). Figure 4B illustrates another instructive case where two true cross-peaks (solid blue squares) are close along both the direct and indirect dimension and only one 1D peak will be predicted for any column (bold black line) and any row (bold red line), despite that the cross-peak shape suggests the presence of two strongly overlapping peaks. In this case, the black and red lines reflecting column-based and row-based 1D peaks, respectively, are tilted deviating significantly from straight vertical and horizontal directions. The 2D peak-picking algorithm searches for this type of pattern by calculating the angle between the most tilted segment of the black and red lines. If both angles are larger than a cutoff of 14°, the peak at the intersection will be replaced by two new peaks, whose positions are defined by the midpoints of the end positions of the two segments. The most tilted segments of the black and red lines are plotted as dotted lines in Fig. 4B and the new cross-peaks as open blue circles. The predicted peaks slightly deviate from the exact locations of the true peaks (filled blue circles), which shows that in the case of such strong peak overlap, the extracted 2D cross-peaks can have some small positional errors.

Our method can be extended from 2D to 3D NMR spectra by analyzing 1D cross-sections along all three dimensions in a manner that is analogous to the extension of DEEP Picker from 1D to 2D. Since DEEP Picker was specifically trained on 1D spectra that are representative for cross-sections of 2D spectra in terms of a number of points per peak, lineshapes, etc., adaptation to 3D spectra, which tend to have much lower digital resolution while suffering from fewer cross-peak overlaps due to the 3rd dimension, poses new challenges. We plan to extend DEEP Picker to 3D (and possibly even higher dimensional spectra) in the future by training a neural network that uses fewer points per peak.

### Application to ^15^N–^1^H HSQC spectra of proteins

**Fig. 5:**
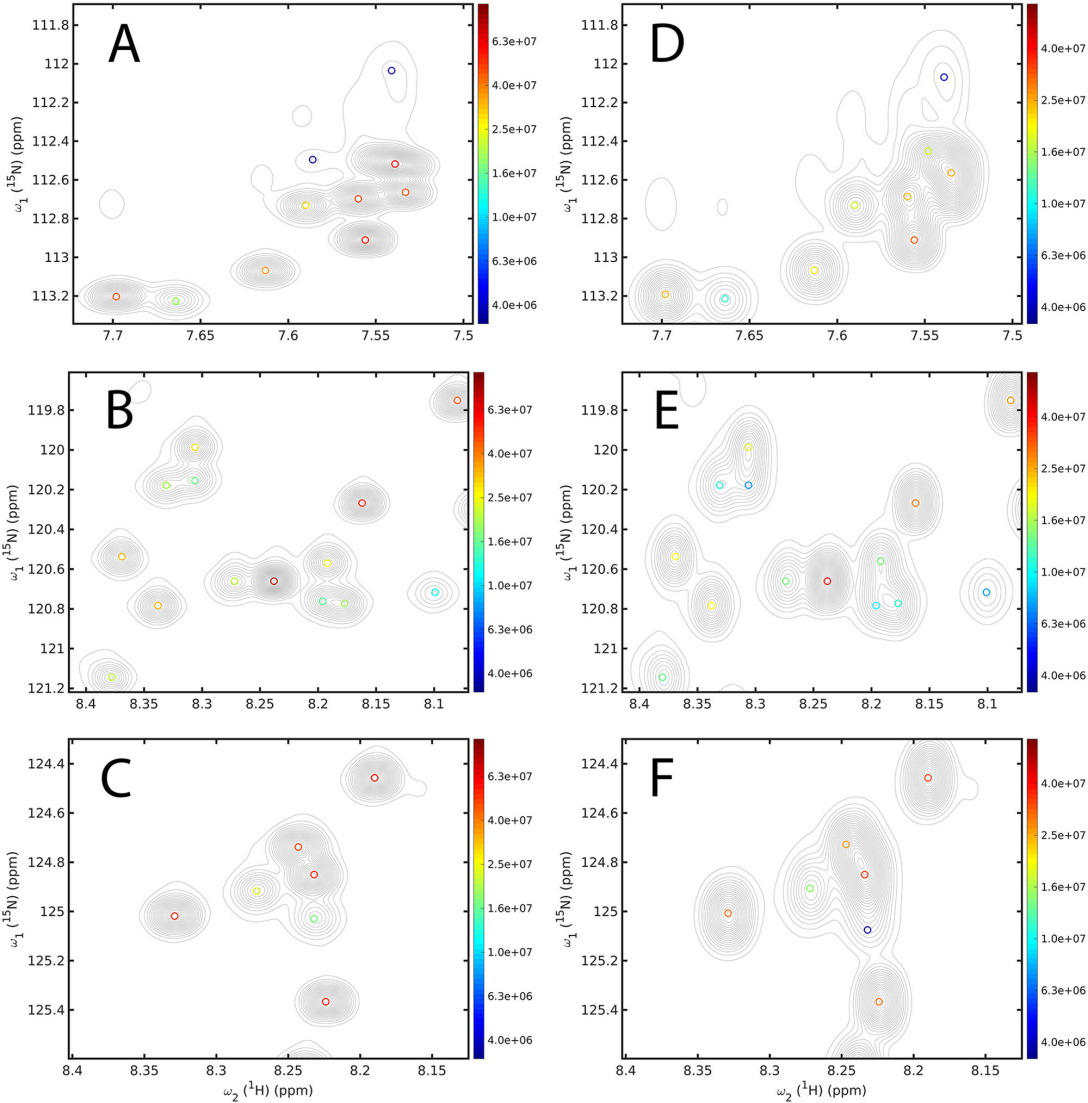
Performance of DEEP Picker for selected regions of 2D ^15^N–^1^H HSQC spectrum of α-synuclein. **A**–**C** HSQC spectrum processed with original resolution and **D**–**F** with reduced resolution along indirect dimension. Three pairs of panels (**A**, **D**), (**B**, **E**), (**C**, **F**) show the same 2D regions for comparison. Picked cross-peaks are indicated as circles and color-coded according to their amplitude on a logarithmic scale, whereas the contour line spacings are linear. Despite the lower spectral resolution in **D**–**F**, DEEP correctly picked the peaks, including all strongly overlapped cross-peaks. Note that the spectra of Panels **D**–**F** have reduced sensitivity since they used only half of the time-domain data.

After training on synthetic data, we applied DEEP Picker to experimental 2D ^15^N–^1^H HSQC spectra of proteins whereby all NMR spectra were processed using NMRPipe^39^ with manual phase correction and automatic polynomial baseline removal. 2D HSQC spectra belong to the most widely used spectra in biomolecular NMR, for example, for fingerprinting, chemical shift perturbation in titration studies, or pseudo-3D NMR experiments for quantitative dynamics studies (*R*_1_, *R*_2_, *R*_1ρ_, CPMG, etc.)^1^. Hence, the accurate computer-assisted analysis of HSQC spectra, including strongly overlapped regions, is important for many different types of NMR applications. We first applied DEEP Picker to α-synuclein, which is an intrinsically disordered 140-residue protein. The ^15^N–^1^H HSQC spectrum was originally measured with 1024 complex data points along the direct dimension and 256 complex data points along the indirect dimension. In order to assess DEEP Picker’s power to recover accurate cross-peak information at high resolution from lower resolution data, we reprocessed the time-domain data by artificially reducing the spectral resolution along the indirect dimension: by removing the *t*_1_ increments 129–256 the spectral resolution was reduced by a factor two. DEEP Picker was then applied to both the original high-resolution and the reduced resolution spectra for comparison. The results for selected regions are shown for the original spectrum in Fig. 5A–C (left panels) and for the reduced resolution spectrum in Fig. 5D–F (right panels). DEEP Picker successfully identified all cross-peaks including those belonging to strongly overlapped regions with the exception of a very low-intensity peak (Fig. 5D) approaching the noise level in the spectrum that used only half of the experimental data. This demonstrates that the peak picker is able to accurately deconvolute such a complex spectrum, even if the resolution is limited, provided that the signal-to-noise of the signals of interest is sufficiently high.

**Fig. 6:**
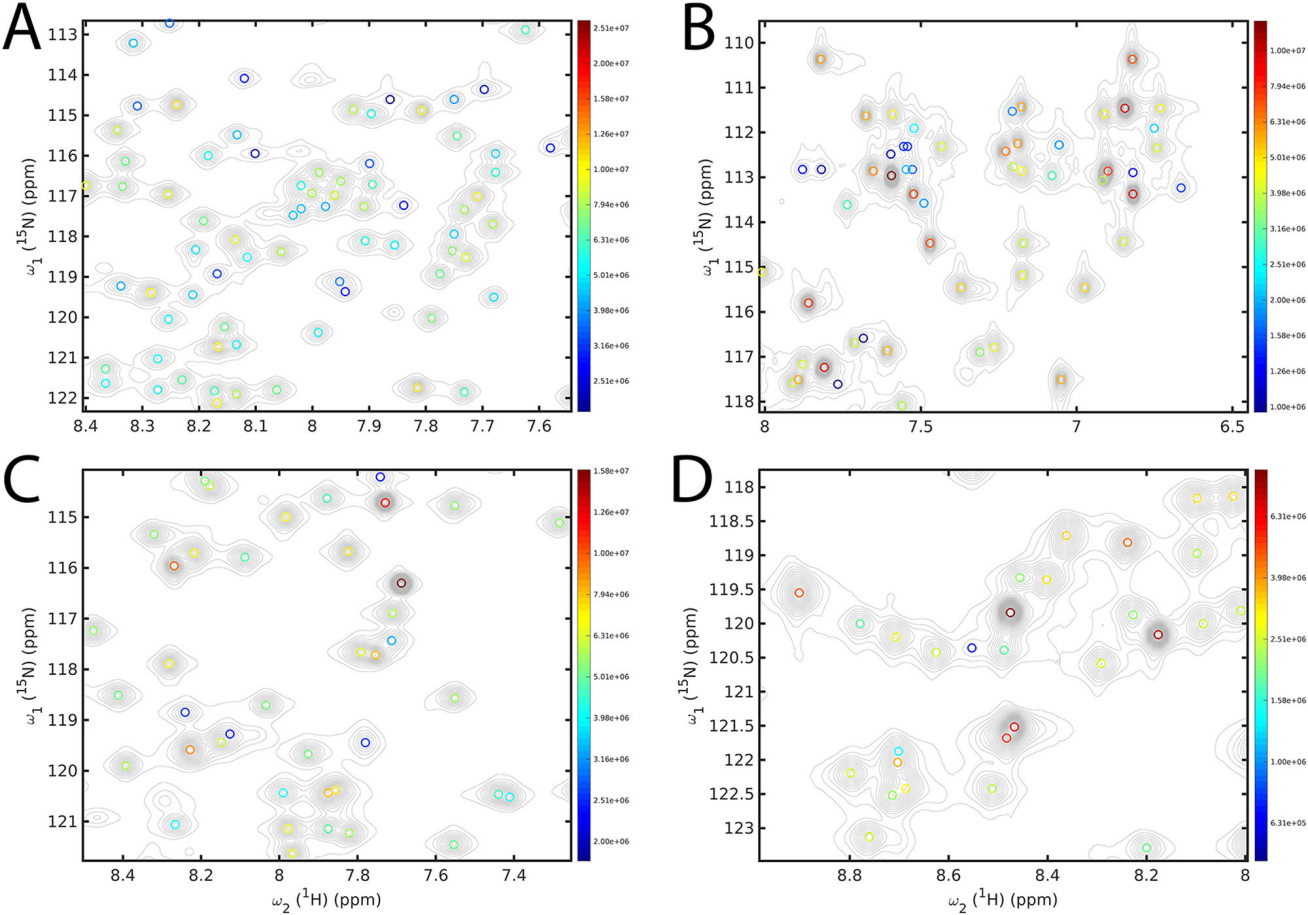
Illustration of peak-picking performance of DEEP Picker for four different proteins. Selected regions of 2D ^15^N–^1^H HSQC spectrum of the four different proteins **A** Gankyrin, **B** PLA2, **C** ARID, and **D** Rop. Picked cross-peaks by DEEP Picker are indicated as circles and color-coded according to their amplitude on a logarithmic scale, whereas the contour line spacings are linear. Experimental information and enlarged plots of each spectrum are given in the Supporting Information. Some of the weakest cross-peaks (small number of contours) were not picked because they are below the noise cutoff used by DEEP Picker.

DEEP Picker works well also for globular proteins as is demonstrated in Fig. 6 for ^15^N–^1^H HSQC spectra of four different proteins, namely Gankyrin^40^ (24.4 kDa), PLA2^41^ (13.8 kDa), ARID^42^ (10.9 kDa), and Rop^43^ (14.2 kDa). All four spectral regions depicted have significant amounts of cross-peak overlap, which are handled by DEEP Picker remarkably well (for additional information, see Supporting Information). Figure S6 shows a comparison of the peak picking results of NMRPipe, Sparky, NMRViewJ and DEEP Picker for challenging regions of protein ^15^N–^1^H HSQC spectra, whereby only DEEP Picker successfully identified all shoulder peaks.

### Application to ^13^C–^1^H HSQC of metabolomics sample

**Fig. 7:**
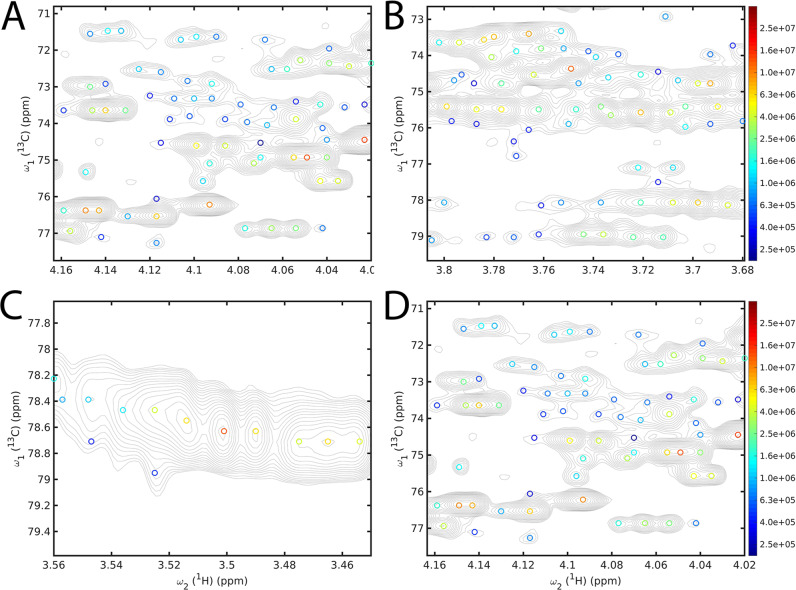
Illustration of performance of DEEP Picker for 2D ^13^C–^1^H HSQC of mouse urine. Selected spectral regions are depicted in Panels **A**–**D**, which include the highly crowded carbohydrate region. DEEP Picker is able to identify and distinguish between cross-peaks that strongly overlap, which poses a significant challenge for their analysis by traditional peak pickers. Picked cross-peaks are indicated as circles and color-coded according to their amplitude on a logarithmic scale with logarithmic contour line spacings.

NMR spectra of metabolomics samples represent another important class of samples where strong peak overlaps can occur in some regions of 2D ^13^C–^1^H HSQC spectra, which are usually measured at ^13^C natural abundance, because of the often large number of different metabolites present in such samples. In contrast to protein NMR spectra, the large dynamic range of peak amplitudes and amplitudes due to large differences in metabolite concentrations pose an additional challenge. A key objective of metabolomics studies is “fingerprinting”, which is the unique identification and analysis of as many cross-peaks as possible, even for ones that barely exceed the noise level, toward a comprehensive and quantitative analysis of these types of biological samples. Because of their small size compared to proteins, metabolites undergo rapid overall tumbling leading to long transverse relaxation times and sharp cross-peaks with small linewidths, but the number of cross-peaks can be very large depending on the complexity of the sample. We demonstrate the application of DEEP Picker for a 2D ^13^C–^1^H HSQC spectrum of mouse urine, which may contain hundreds of different metabolites with various concentrations. Selected spectral regions of the spectrum together with the picked cross-peaks are shown in Fig. 7A–D. The aliphatic regions shown belong to some of the most crowded regions of urine spectra that include numerous carbohydrates. Because a dominant fraction of the cross-peaks of mouse urine belongs to unknown metabolites, the ground truth is largely unknown. Hence, Fig. 7 primarily serves as an illustration of the performance of DEEP Picker. Nonetheless, visual inspection shows how DEEP Picker is able to identify and distinguish between strongly overlapping cross-peaks that pose significant challenges for their analysis from standard 2D ^13^C–^1^H HSQC experiments^44^. More accurate spectral analysis directly benefits the identification of metabolites in urine and other complex metabolomics mixtures, which is a key step toward their quantitative profiling^2^.

### Application to NOESY and TOCSY spectra

**Fig. 8:**
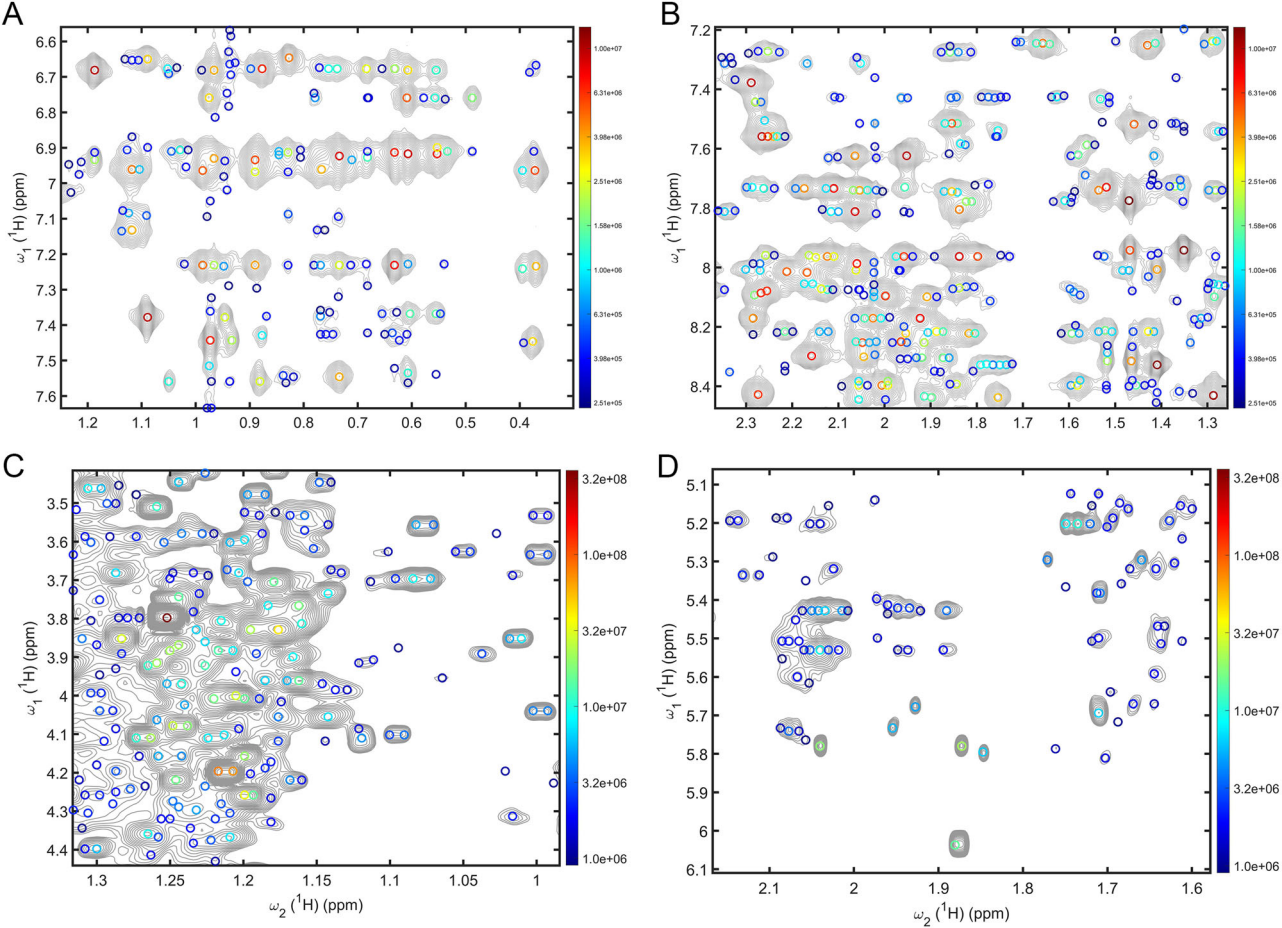
Application of DEEP Picker to 2D NOESY and TOCSY spectra. **A**, **B** Selected regions of 2D ^1^H–^1^H NOESY of Im7 and **C**, **D** 2D ^1^H–^1^H TOCSY of mouse urine with picked cross-peaks indicated as circles that are color-coded according to their amplitude (logarithmic scale, see sidebar). DEEP Picker identifies strong and weak cross-peaks, including ones that severely overlap or show multiplet structures due to J-splittings, whose analysis is often challenging for traditional peak pickers.

When applied to other common 2D NMR experiments, such as NOESY and TOCSY, which tend to possess a larger dynamic range along with larger numbers of challenging peaks than HSQC spectra, DEEP Picker does a remarkable job too. This is demonstrated in Fig. 8, which shows regions of a NOESY spectrum of protein Im7 and a TOCSY spectrum of urine. DEEP Picker is able to identify also individual multiplet components due to J-splittings, which can be challenging for traditional peak pickers. DEEP Picker has generally a higher confidence score for major cross-peaks and lower confidence in low amplitude cross-peaks or multiplet components. Selected regions of the NOESY spectrum with picked peaks that are color-coded according to their confidence level score are shown in Fig. S8. Similar to Fig. 7, since the ground truth of NOESY and TOCSY spectra with their very large number of cross-peaks is only partially known, Fig. 8 serves primarily as an illustration of what can be expected of DEEP Picker for such kind of complex spectra.

### Quantitative performance and effect of noise and other artifacts

A quantitative and objective assessment of a peak picker is desirable. However, unlike other common machine learning applications, there is no large, carefully curated NMR spectral test database available for an objective assessment of NMR peak-picking performance. Here, we used previously determined or published cross-peak assignments that were obtained with the help of complete sets of 3D assignment experiments. We assessed the picked ^15^N–^1^H HSQC cross-peaks in terms of the number of false negatives and false positives, whereby “false” positives were visually inspected as they may correspond to true cross-peaks belonging to impurities, chemically modified, or aggregated proteins. In Table S2, quantitative statistics and performance metrics of DEEP Picker are compiled for two of the most challenging proteins described here. The results suggest that the accuracy of DEEP Picker is very high with the only false negative peaks corresponding either to peaks that almost perfectly overlap with other peaks in the 2D ^15^N–^1^H HSQC and could only be identified with the help of additional 3D triple-resonance (^1^H, ^13^C, ^15^N) NMR experiments or because they were weak falling well below a given amplitude cutoff (see Supporting Information). Five peaks with high amplitudes were identified by DEEP Picker in both protein spectra that upon visual inspection look like real peaks, but had not been assigned. A large number of weak peaks were identified by DEEP Picker with amplitudes <10% of the major cross-peaks that had been previously assigned. Visual inspection, based on contour plots with the lowest contours drawn at a very low level, revealed that these cross-peaks are in all likelihood true peaks (Fig. S9). Their unambiguous annotation as main peaks requires spin connectivity information from additional multi-dimensional NMR experiments, but due to their low amplitudes sensitivity could be a significant challenge. It is possible that, in addition, certain noise artifacts or peaks with small phase errors are computationally indistinguishable from true peaks. Since their amplitudes are usually only a fraction of the major peaks that are of primary interest for the vast majority of NMR applications, they can be effectively filtered out during post-analysis using cutoff criteria based on amplitude.

Although the HSQC spectra used in this work stem from “real-world” applications with signal-to-noise ratios (S/N) that are typical for samples measured at our shared NMR facility, we measured additional HSQC spectra on a K-Ras sample with a concentration of only 130 μM. We collected a 30 m ^15^N–^1^H HSQC spectrum with only four scans per increment and, for comparison, also with 108 scans per *t*_1_-increment improving S/N by over a factor of 5. As shown in Fig. S7, the application of DEEP Picker reveals that even for the low-sensitivity spectrum with S/N ≅ 25:1, DEEP picks all isolated peaks correctly and is able to identify the vast majority of shoulder peaks. Sometimes, however, multiple peaks are picked around a peak maximum because of the uneven peak shapes displayed by the noisy spectrum, and some low-amplitude peaks close to the noise floor are lost.

Because DEEP Picker uses local information only, artifacts or noise that share the same local features with true peaks cannot be easily recognized. Such artifacts are best identified and removed in a column-by-column post-analysis, including residual water signals that have a well-defined ^1^H chemical shift or *t*_1_-noise forming vertical signal streaks along the indirect dimension, and they were not counted as false positives. Similarly, phase errors of the input spectrum are best identified by inspection of the entire spectrum rather than based on individual peaks. They are manifested as minor peaks associated with main peaks in a systematic uniform (0th order) or frequency-dependent (1st order) manner and they are best removed by reprocessing the original spectrum. On the other hand, if only selected peaks possess such features, they correspond most likely to true overlapped peaks and should be kept in the final peak list. A residual smooth baseline artifact, a small phase error (<2°), or slightly imperfect peak shapes caused, e.g., by temperature fluctuations or shimming issues, are generally tolerated by DEEP Picker, since in our training set we intentionally annotated some very closely overlapped peak pairs as a single peak. On the other hand, DEEP Picker was not designed for the analysis of very low-quality spectra exhibiting peaks with significant distortions or substantial amounts of truncation artifacts. A neural network could be trained to recognize truncation artifacts but not for the discrimination between real peaks and noise of similar amplitude.

## Discussion

In the past, Kernel filter methods have been widely used in image classification long before machine learning entered the field. They include specialized filters, for example, for edge and corner detection, sharpening, and blurring^45^. Among these filters, Laplacian-type filters have been proposed for NMR applications as they amplify curvature and certain high-frequency features of a signal^46^. In this way, shoulder peaks can be transformed into local maxima facilitating their identification. In practice, Laplacian filters are often combined with data smoothing to mitigate the effect of noise amplification, which typically results in lower resolution^47^. Laplacian filters might also introduce other artifacts that are indistinguishable from true peaks. For their successful application to real-world spectra, the Laplacian and smoothing filter parameters need to be manually fine-tuned and criteria have to be defined for the removal of artifact peaks. By contrast, convolutional neural networks (CNN) as used for DEEP Picker are well known for their ability to develop their own customized filters from suitable training datasets^48^. From a more qualitative perspective, the first few neural network layers of DEEP Picker represent a host of different filters responsible for edge detection, corner detection, Laplacian-type sharpening, and denoising. Additional layers are trained to detect higher-level abstract features. All this information is then used to decide which data point is a peak in order to reproduce the ground truth of the training set without any human intervention.

Both the quantity and the quality of the training data are vital for the success of the development of a neural network. Unlike a previous ANN-based peak picker^29^ that was trained using real experimental datasets annotated by human experts, we exclusively rely on synthetic datasets for two reasons. First, the synthetic data can be efficiently generated at almost arbitrary amounts and diversity so that the optimal complexity of the neural network was not limited by the amount of available training data. Second, we could easily curate the training data to ensure satisfactory class balance. For a suitable 1D peak training set, we need a similar number of peaks in each of the following categories independent of the actual frequency of these features in experiments: standalone peaks, peaks with a shoulder peak on the left-hand side of the main peak, peaks with a shoulder peak on the right-hand side of the main peak, peaks with two shoulder peaks on both sides, etc. In practice, a substantial number of synthetic spectra were generated, followed by the trimming of overrepresented classes to satisfy the class balance. Such class balance is much easier to achieve in synthetic datasets compared to experimental sets. In the latter case, the many possible scenarios of strongly overlapped peaks are usually significantly undersampled. In the case of 2D spectra, even when using synthetic datasets, coverage of the many possible overlap scenarios was still a challenge. To address this problem, we developed a hybrid approach by applying the deep neural network peak picker to all 1D cross-sections of the 2D spectrum along both frequency dimensions *ω*_1_ and *ω*_2_ followed by the use of a traditional decision table to identify the 2D cross-peaks based on the 1D cross-section results. This approach turned out to be remarkably accurate and robust for very different NMR samples, including folded proteins, intrinsically disordered proteins, and a highly complex urine metabolomics sample.

For the development of a useful and versatile peak picker, a crucial task is to draw the decision boundary between single peaks and two (or more) overlapped peaks as illustrated in Fig. 1C–F. Not surprisingly, such a decision boundary could be defined without ambiguity only when the spectrum is noise-free and all peaks followed the Voigt profile without any artifacts (phasing or baseline errors). For real-world spectra, it is up to the spectroscopist to define the decision boundary, by taking the quality of the input spectrum, the allowed line shapes of all peaks, etc., into consideration. During the peak deconvolution process, nonlinear peak fitting provides a quantitative metric for how well the input spectral region can be explained by a single peak, for example, using either the root-mean-square fitting errors or the maximal absolute error as a metric. We employed nonlinear peak fitting to provide the ground truth for spectral labeling using a mathematically defined decision boundary. We can achieve consistency across all training samples, which is another distinction from previous methods that mostly relied on labeling by human experts. The threshold of 3% (see the “Results” section) was selected in this work to best reproduce the consensus among a group of NMR experts in our lab. One can obtain a more sensitive neural network by generating training samples with a smaller threshold. Such a new peak picker might perform better for spectra with very high signal-to-noise ratios and uniform peak lineshapes. On the other hand, a less sensitive neural network might be more suitable for low signal-to-noise spectra or spectra with variable peak lineshapes.

It is worth emphasizing that DEEP Picker predicts every peak locally without taking into account the behavior of spectral data points that are further away. To further improve the consistency of all the predicted peaks, especially for the overlapped peak clusters, one can run a non-linear least-squares fit of all peaks simultaneously, using the results returned by DEEP Picker as a starting point. Because non-linear least-square peak fitting cannot guarantee that the identified *χ*^2^ minimum is the global minimum, the quality of the starting point is vital for the best results. Based on our experience with DEEP Picker, we found that it provides under many different circumstances an excellent starting point for the fully quantitative fitting of the peak parameters. Like any other neural network, DEEP Picker works best for applications that are overall similar to the datasets it has been trained for. A requirement is that all peaks in the input spectra must have in good approximation a Voigt profile, which can be achieved in practice quite easily, for example, by using the Kaiser window apodization function during Fourier transform processing. Finally, the spectral resolution must be sufficiently high so that each peak is represented by 6–20 (or 4–12) points, which is readily achievable by the application of a proper amount of zero-filling during processing.

DEEP Picker is a fast, versatile, and highly accurate peak picker as demonstrated here for HSQC spectra, which are among the most widely used 2D NMR spectra of proteins and metabolomics applications providing powerful spectral fingerprints of complex molecular systems. The machine-learning-based DEEP spectral analyzer was developed solely based on synthetic data that closely mirror peak shapes and linewidths encountered in NMR experiments but without any analytical mathematical guidance. DEEP Picker is able to reproducibly deconvolute spectral regions with severe peak overlaps providing more complete access to the valuable information contained in these types of NMR spectra. For this reason, DEEP Picker is now the default peak picking and peak fitting engine of our COLMAR suite of metabolomics webservers^49^. DEEP Picker is expected to play a useful role toward increasing automation and standardization of NMR data processing protocols to make the results both unequivocally and easily transferable between different spectrometers, projects, and research labs.

### Reporting summary

Further information on research design is available in the Nature Research Reporting Summary linked to this article.

## Supplementary information

Supplementary Information

Reporting Summary

## Data Availability

Spectra of α-synuclein, Im7, and urine along with peak annotation generated by DEEP Picker is available in the Zenodo database (https://zenodo.org/record/5155575#.YQlF6o5KgbU)^50^.

## Code availability

The training script based on TensorFlow v1.3 and The C/C++ implementation of DEEP Picker is freely available from our website http://spin.ccic.osu.edu/index.php/download and GitHub (https://github.com/lidawei1975/deep)^51^ under the GNU General Public License Agreement.
