# Supplementary material for: DEEP picker is a deep neural network for accurate deconvolution of complex two-dimensional NMR spectra
Source: Nat Commun. 2021 Sep 1;12:5229. doi: 10.1038/s41467-021-25496-5 (PMC8410766; doi:10.1038/s41467-021-25496-5)
Supplement: Supplementary file 1 — Supplementary Information [file 41467_2021_25496_MOESM1_ESM.pdf]

Supporting Information

DEEP Picker is a Deep Neural Network for Accurate Deconvolution of Complex Two-Dimensional NMR Spectra

Da-Wei Li,[1*] Alexandar L. Hansen,[1] Chunhua Yuan,[1] Lei Bruschweiler-Li[1] and Rafael Brüschweiler[1,2,3*]

[1]Campus Chemical Instrument Center, The Ohio State University, Columbus, Ohio 43210, USA

[2]Department of Chemistry and Biochemistry, The Ohio State University, Columbus, Ohio 43210, USA

[3]Department of Biological Chemistry and Pharmacology, The Ohio State University, Columbus, Ohio 43210, USA

Automated spectral noise level estimation

We define the noise level of an NMR spectrum as 1.485 times the median absolute deviation (MAD) of the full spectrum by performing two rounds of iterations after signals that exceed the threshold have been removed after the first iteration. This measure is notably robust as long as there are sizeable empty regions in the spectrum as is the case for the 2D spectra studied here.

DEEP Picker uses a "low peak amplitude cutoff" (LPAC) to consider an identified feature as a peak, which depends on the molecular system and the intended use of the spectrum. For protein ^{15}N-^{1}H HSQC applications, the default LPAC is 9 x noise level, whereas for metabolomics the default cutoff is 5.5 x noise level. LPAC can be adjusted by the user or the returned peak list can be edited according to amplitude as well as other criteria specified by the user during post-processing. The optimal LPAC also depends on the signal-to-noise-ratio of the spectrum, the dynamic range of cross-peaks of interest for downstream analysis, and the presence of sample impurities, chemically modified, or aggregated proteins. A future goal will be the establishment of rules for an optimal choice of the LPAC for various types of applications, which should be possible by analyzing spectra of many different biomolecular systems with DEEP Picker.

Table S1. List of proteins and 2D spectra analyzed using DEEP Picker with experimental conditions.

Abbreviated protein/ sample name	K-Ras	aSyn	Im7	PLA2	ARID	Rop	Gankyrin	Urine	Urine
Full name	G-domain of K-Ras G12C mutation	α-synuclein	Colicin E7 Immunity Protein	Bovine pancreatic phospholipase A2	AT-rich interaction domain in the histone H3 lysine 4 demethylase RBP2	Repressor of primer	Human oncogenic protein Gankyrin	Mouse urine	Mouse urine
Number of residues	169	140	87	123	98	63x2 (homo-dimer)	226	n.a.	n.a.
Molecular weight (kDa)	19.5	14.5	10.0	13.8	10.9	14.2	24.4	n.a.	n.a.
Temperature	298 K	298 K	298 K	310 K	293 K	298 K	300 K	298 K	298K
pH	7.0	7.0	7.0	6.0	6.0	6.3	7.4	7.4	7.4
NMR B_0 field (^{1}H frequency)	850 MHz	850 MHz	850 MHz	600 MHz	600 MHz	600 MHz	800 MHz	850 MHz	850 MHz
Type	HSQC	HSQC	NOESY	HSQC	HSQC	HSQC	HSQC	HSQC	TOCSY
TD2 (real pts)	4096	2048	4096	2048	1024	1024	2048	2048	4096
TD1 (real pts)	256	512	720	256	256	128	256	512	1024
N2 (real pts)	8k	8k	16k	8k	4k	4k	8k	8k	8k
N1 (real pts)	2k	2k	2k	1k	1k	512	1k	2k	2k
Literature reference of HSQC spectra	new data	new data	new data	Ref.[1]	Ref.[2]	Ref.[3]	Ref.[4]	Ref.[5]	new data

Table S2. Quantitative assessment of DEEP Picker performance for selected 2D ^{15}N-^{1}H protein NMR spectra.

Protein	Number of assigned peaks[a]	Number of missed peaks by DEEP[b]	Number of false negatives	Number of major, unassigned peaks picked by DEEP[c]	Number of false positive peaks picked by DEEP	Number of picked peaks with amplitude below LPAC[d]	Setting of LPAC as a multiple of noise level[e]
K-Ras	166	2, 1	0	5	0	215	40
Gankyrin	217	2, 1	0	5	0	158	20

[a] 2D ^{15}N-^{1}H HSQC peaks that were previously assigned with the help of sets of 3D triple-resonance (^{1}H, ^{13}C, ^{15}N) NMR experiments and correctly identified by DEEP Picker with the exception of the peaks listed in subsequent columns of the table. ^{15}N-^{1}H HSQC cross-peaks of Asn and Gln side-chain resonances and peaks with ^{1}H frequencies at the same position as the water resonance were excluded. These cross-peaks fall in well-defined and well-known spectral regions and, hence, could be easily identified and excluded from analysis.

[b] Peaks that can only be identified with the help of 3D experiments because of severe overlap in the 2D ^{15}N-^{1}H HSQC spectrum with another cross-peak (first number) or because the peak amplitude is below the low peak amplitude cutoff (LPAC) (second number).

[c] Major peaks picked by DEEP Picker, which are unassigned but are deemed to be true peaks by visual inspection. An example of such a peak is shown in Figure S9.

[d] These are cross-peaks picked by DEEP Picker with amplitudes much lower than those of the major cross-peaks of the spectra. As illustrated in Figure S9, these weak cross-peaks appear, as judged by visual inspection, to be true peaks identified by DEEP Picker. They are likely to stem from impurities, chemically modified, or aggregated proteins and are not further pursued in most biomolecular NMR studies.

[e] For NMR spectra with high signal-to-noise-ratios, as is the case for K-Ras, the optimal LPAC is set at a higher level in order to filter out unwanted low-amplitude cross-peaks.

Table S3. Weights of the different terms in the total loss (training target)

Terms	Cross-entropy of non-peak	Cross-entropy of class 2 peak	Cross-entropy of class 1 peak	MSE of class 2 peak parameters	MSE of class 1 peak parameters
Weights	0.4	1.4	0.9	5.0	15.0

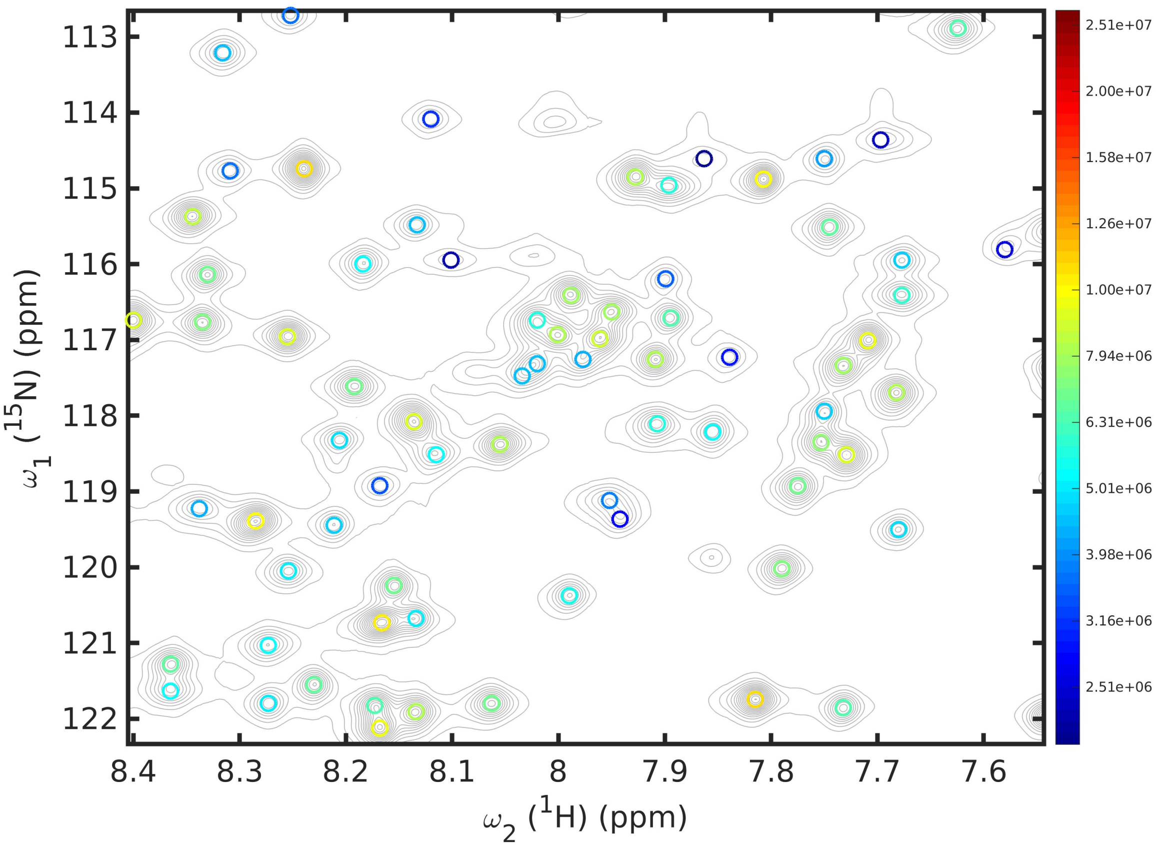

Figure S1. Selected region of ^{15}N-^{1}H HSQC spctrum of Gankyrin. Contour lines are plotted using a linear scale and the cross-peaks picked by DEEP Picker are indicated by an open circle that is color-coded according to the cross-peak amplitude on a logarithmic scale (see color sidebar). LPAC was set to 40 times and the lowest contour level at 15 times the noise level.

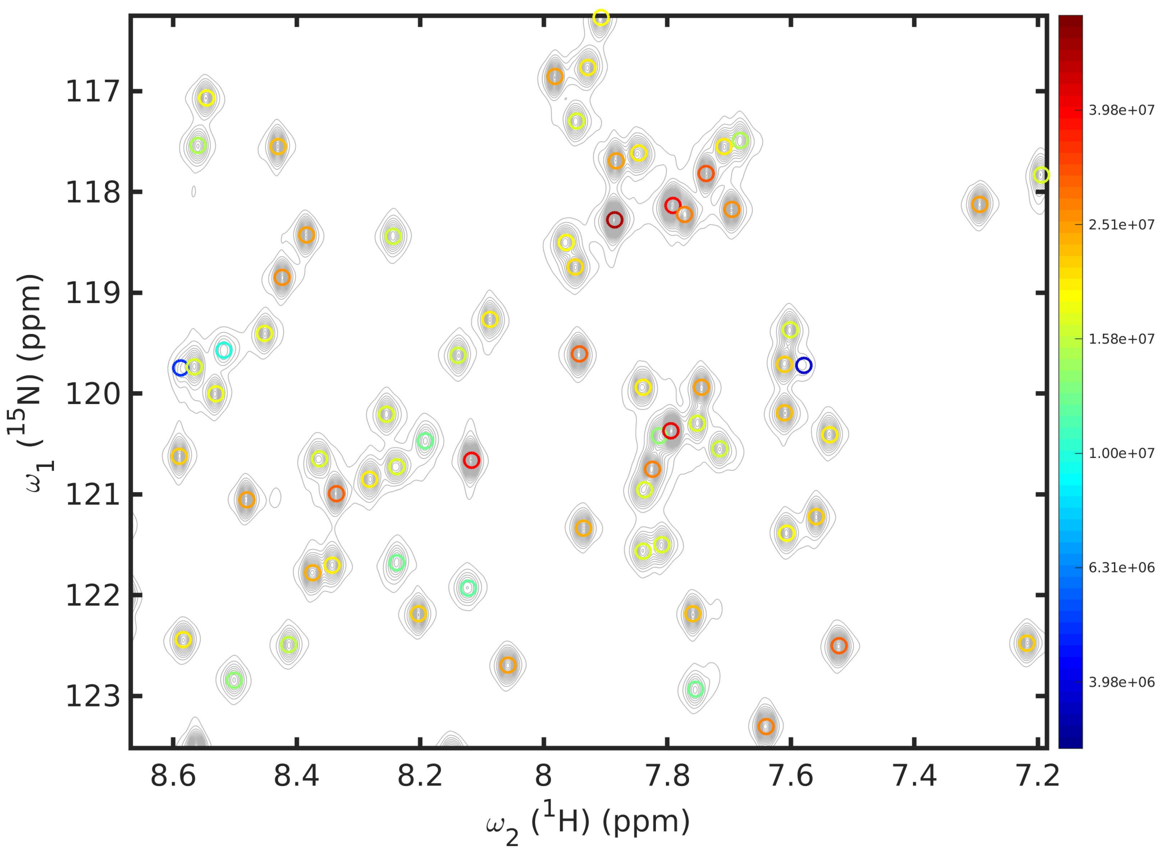

Figure S2. Selected region of ^{15}N-^{1}H HSQC spectrum of K-Ras protein. Contour lines are plotted using a linear scale and the cross-peaks picked by DEEP Picker are indicated by an open circle that is color-coded according to the cross-peak amplitude on a logarithmic scale (see color sidebar). LPAC was set to 35 times and the lowest contour level at 15 times the noise level.

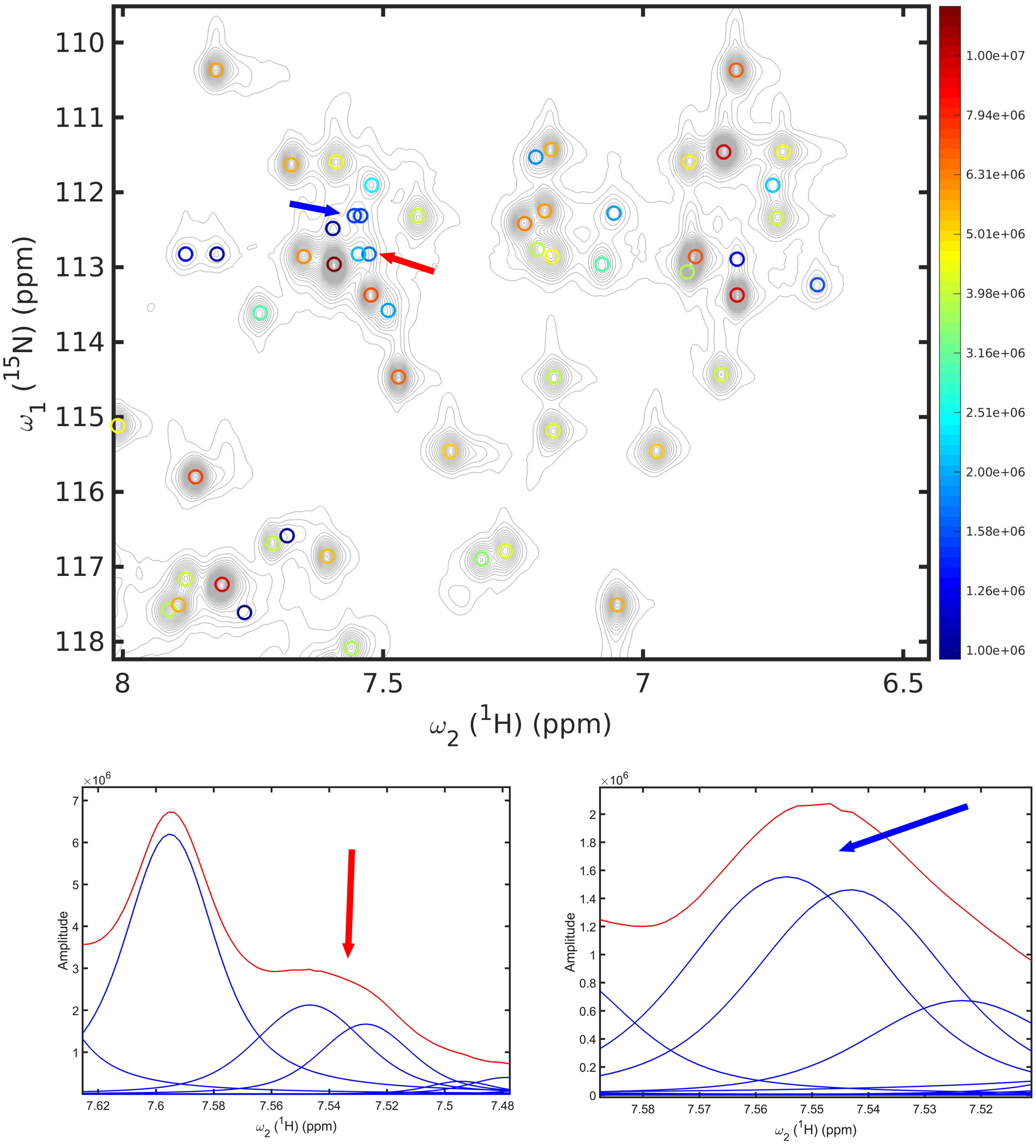

Figure S3. Selected region of ^{15}N-^{1}H HSQC spectrum of PLA2 protein. Contour lines are plotted using a linear scale and the cross-peaks picked by DEEP Picker are indicated by open circles that are color-coded according to the cross-peak amplitude (on a logarithmic scale, see color sidebar). In the vicinity of major peaks, DEEP Picker sometimes picked multiple peaks (labeled with blue and red arrows). Closer inspection of the 1D cross-sections, as shown in the two bottom panels, shows that these peaks are picked correctly. In the bottom panels, red lines correspond to the input spectra and the blue lines correspond to all deconvoluted 1D peaks returned by DEEP Picker including peaks below the LPAC. LPAC was set to 24 times and the lowest contour level at 6 times the noise level.

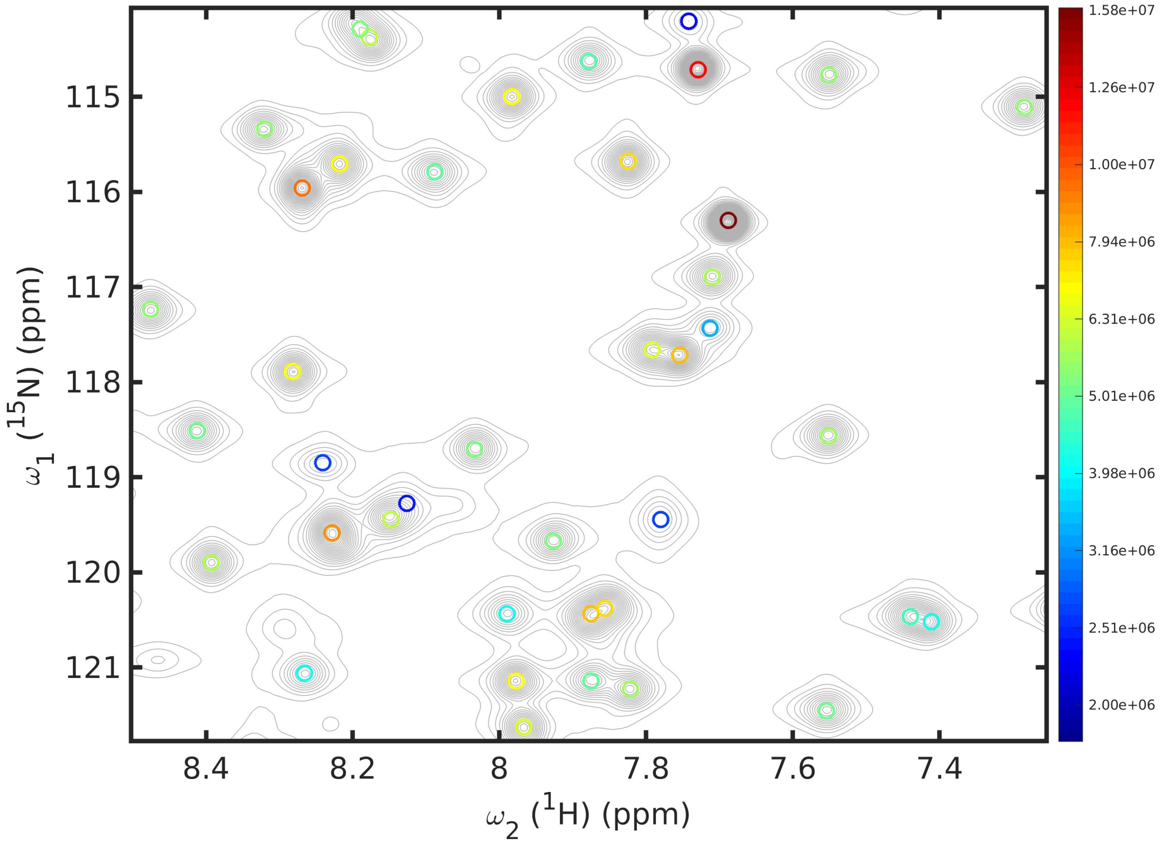

Figure S4. Selected region of ^{15}N-^{1}H HSQC spectrum of the ARID protein. Contour lines are plotted using a linear scale and the cross-peaks picked by DEEP Picker are indicated by an open circle that is color-coded according to the cross-peak amplitude on a logarithmic scale (see color sidebar). LPAC was set to 90 times and the lowest contour level at 20 times the noise level.

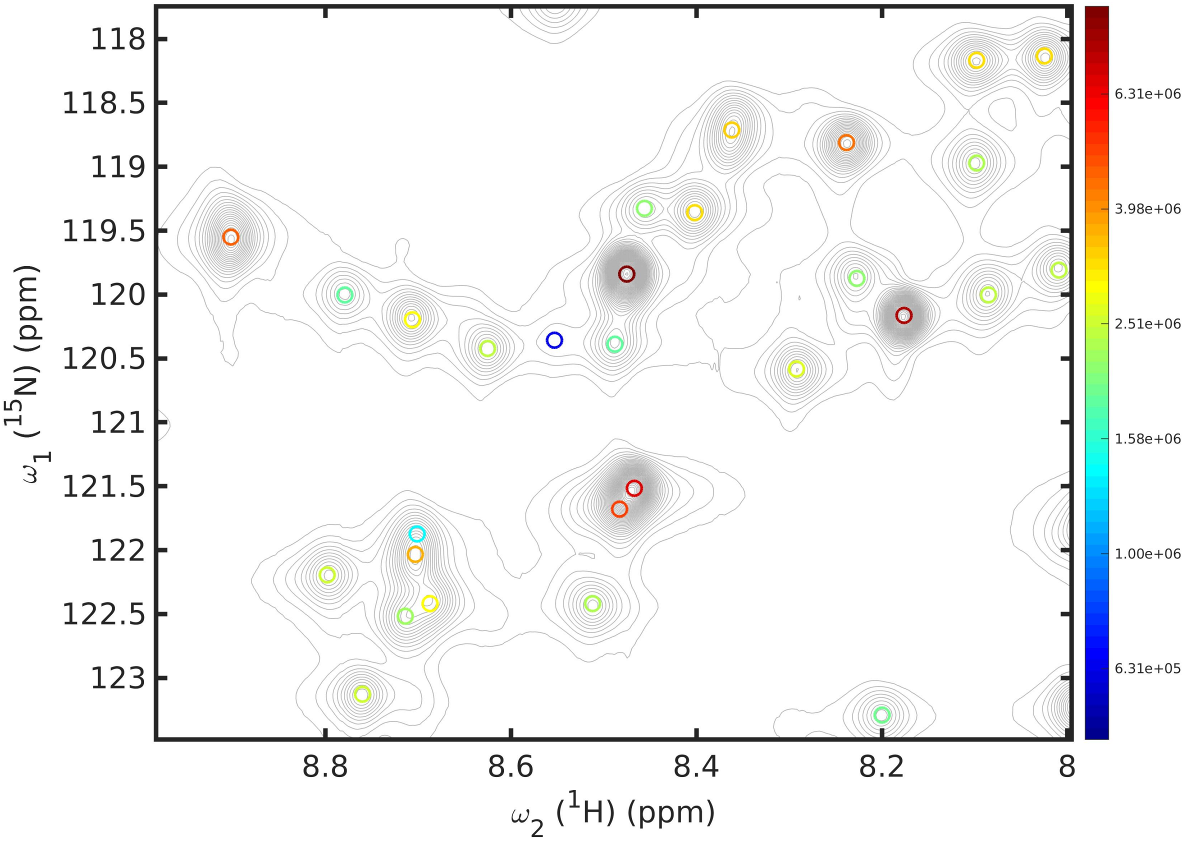

Figure S5. Selected region of ^{15}N-^{1}H HSQC spectrum of Rop protein. Contour lines are plotted using a linear scale and the cross-peaks picked by DEEP Picker are indicated by an open circle that is color-coded according to the cross-peak amplitude on a logarithmic scale (see color sidebar). LPAC was set to 30 times and the lowest contour level at 9 times the noise level.

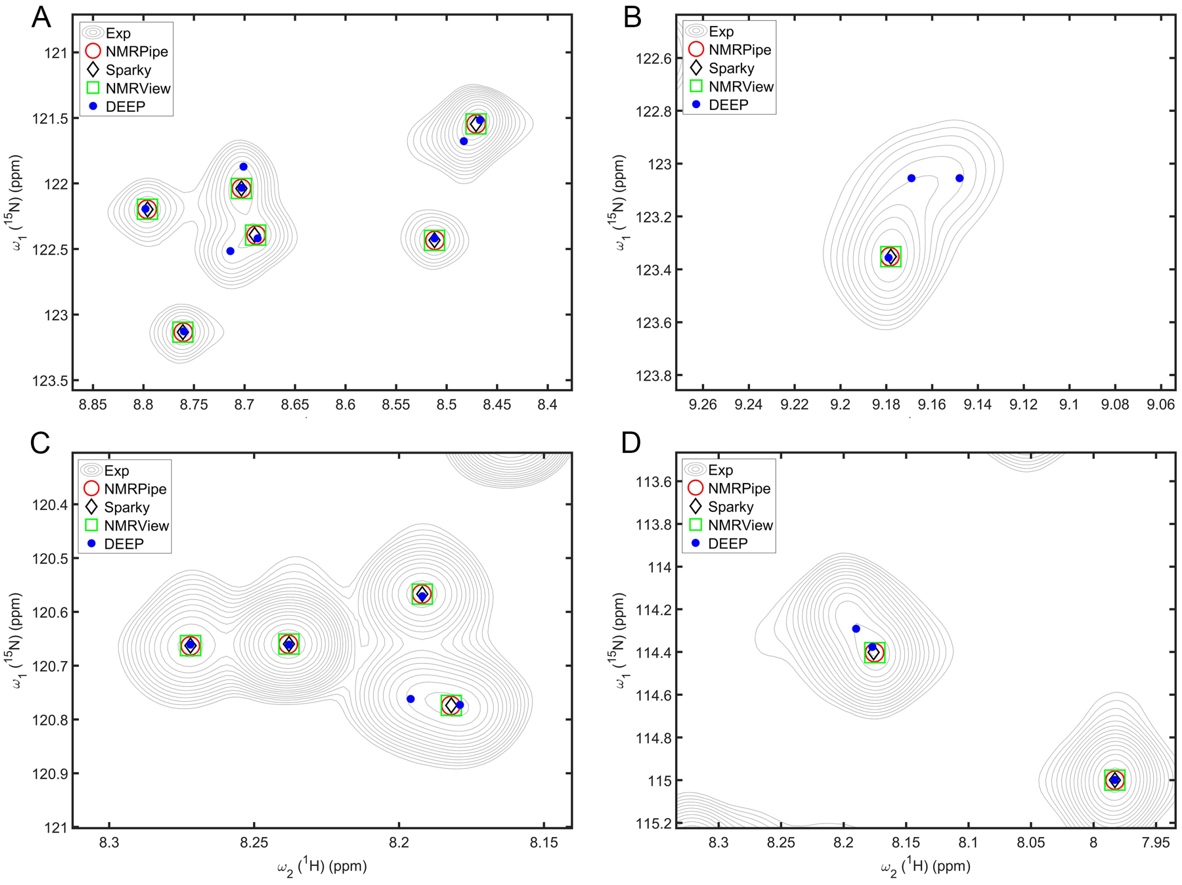

Figure S6. Comparison of peak-picking results of DEEP Picker with the commonly used NMR peak pickers NMRPipe, Sparky, and NMRView. Selected regions of ^{15}N-^{1}H HSQC spectra of proteins belong to (A) Rop, (B) Gankyrin, (C) aSyn, and (D) ARID. Contour lines are plotted using a logarithmic scale. Only DEEP Picker is able to identify all shoulder peaks, including strongly overlapped ones, such as the one in Panel A at (8.48, 121.6) ppm.

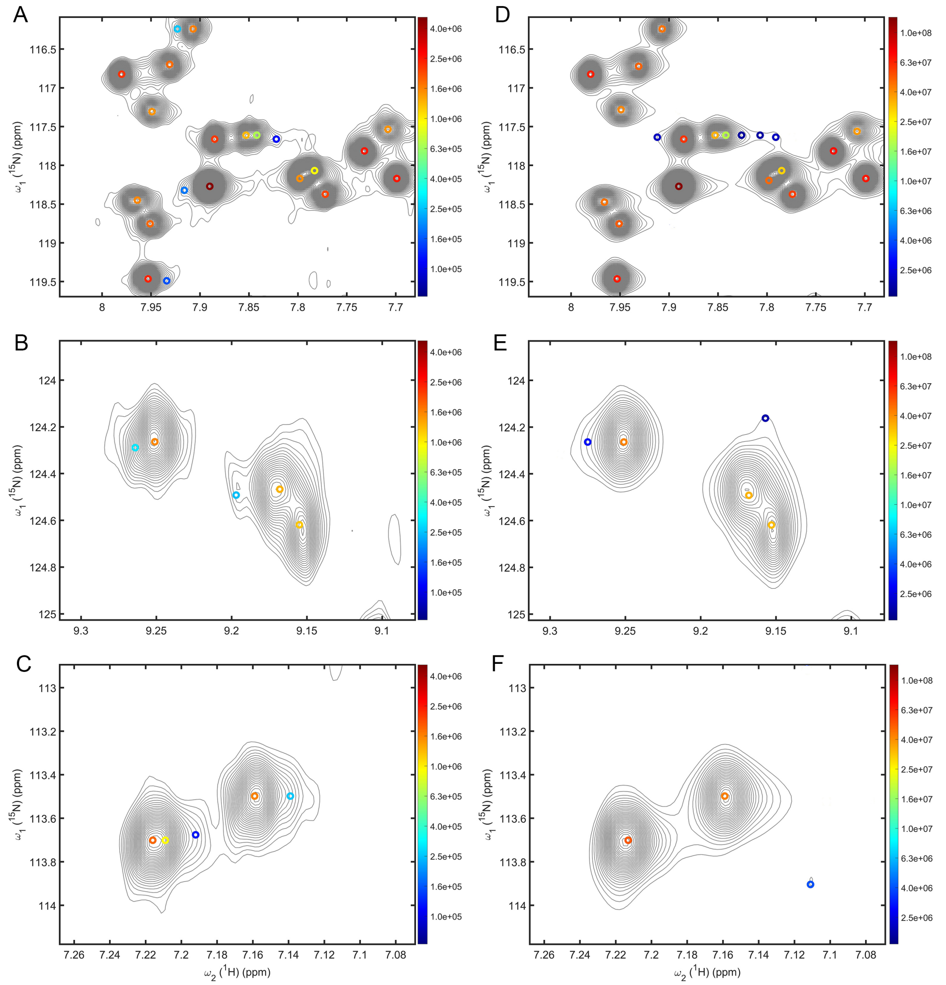

Figure S7. Comparison of the performance of DEEP Picker for the same spectra with different signal-to-noise (S/N). Selected regions of ^{15}N-^{1}H HSQC spectra of K-Ras protein at a very low concentration of 130 μM recorded at 850 MHz proton frequency with either 4 scans (Panels A, B, C) or 108 scans per t_1-increment (Panels D, E, F). Estimated signal-to-noise ratios are 25 (Panels A, B, C) and 125 (Panels D, E, F). Contour lines are plotted using a linear scale and the cross-peaks picked by DEEP Picker are indicated by open circles that are color-coded according to the cross-peak amplitudes (on a logarithmic scale, see color sidebar). In the case of the low S/N spectrum, DEEP Picker sometimes picks multiple peaks on the top of cross-peaks, since the peaks have uneven shapes caused by the presence of noise. On the other hand, DEEP Picker sometimes misses low amplitude cross-peaks when they are too close to the noise floor (see Panels C vs. F). It should be noted that the examples above were chosen specifically to illustrate potential challenges for spectra with low S/N ratios. At the same time, many cross-peaks are picked by DEEP Picker without difficulties even in the low S/N spectrum (see Panels A and B).

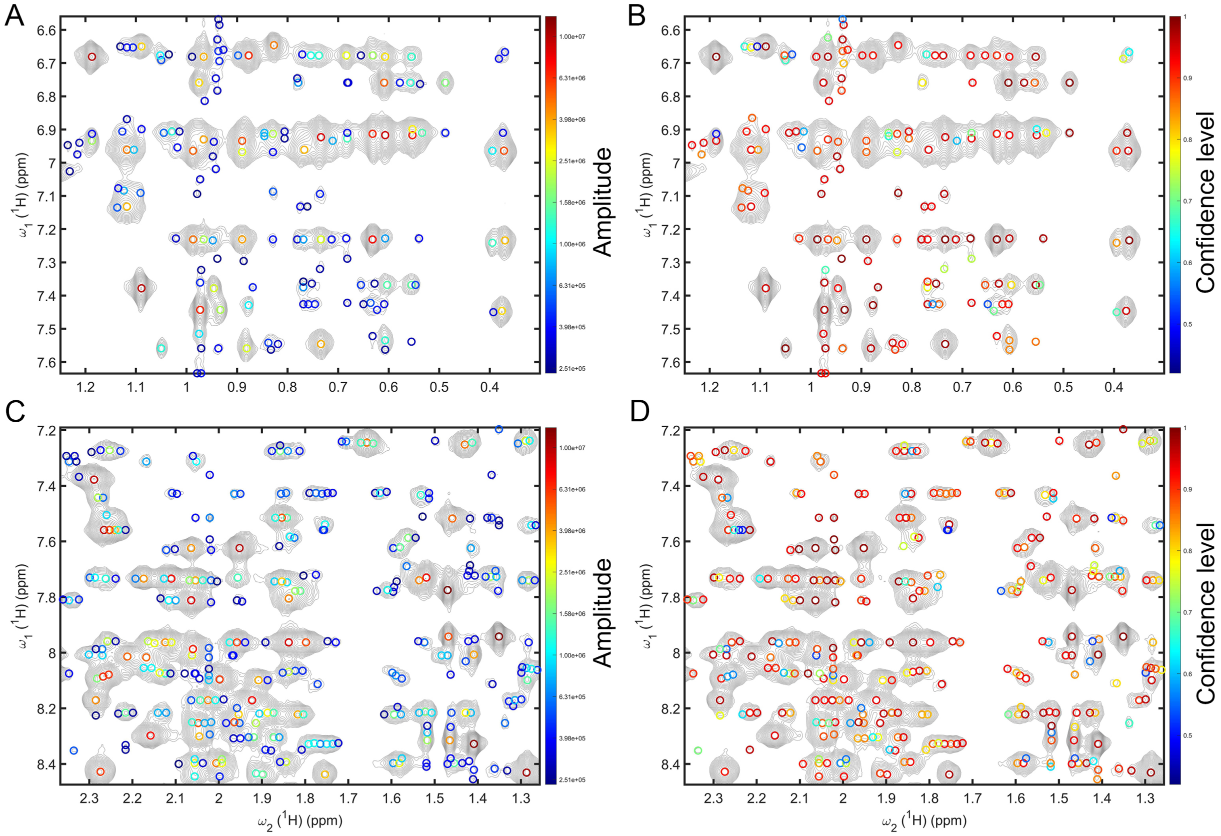

Figure S8. Performance of DEEP Picker for selected regions of 2D NOESY spectrum of protein Im7. Panels A,B and C,D show the same regions, but picked peaks have different color coding. Contour lines are plotted using a logarithmic scale. In Panels A and C, picked peaks are color coded according to the cross-peak amplitude (on logarithmic scale, see sidebar), whereas in Panels B and D, picked peaks are color-coded according to the predicted confidence level score (on a linear scale, see sidebar).

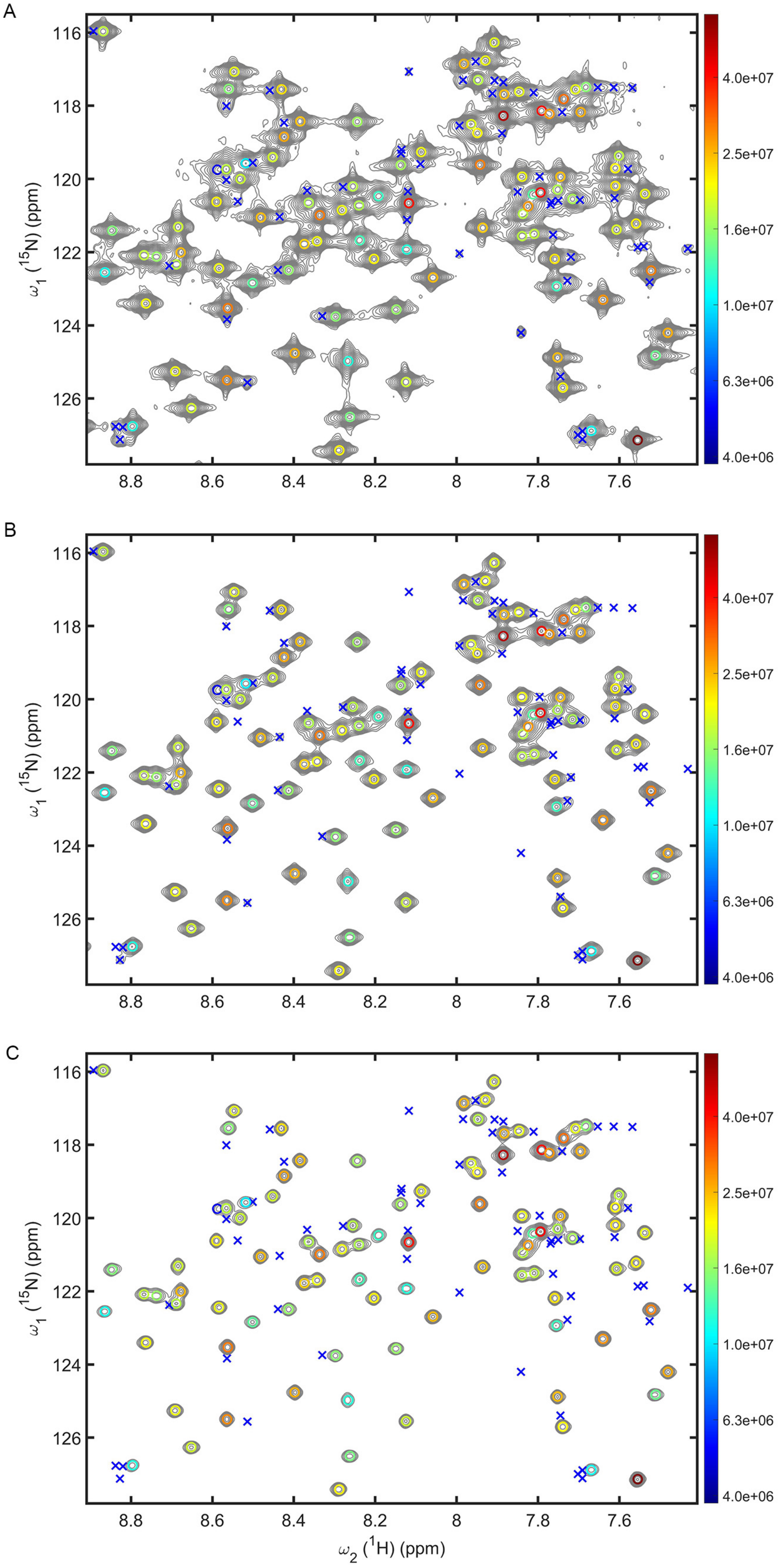
A
B
C
ω1 (15N) (ppm)
ω2 (1H) (ppm)
116
118
120
122
124
126
8.8
8.6
8.4
8.2
8
7.8
7.6
4.0e+07
2.5e+07
1.6e+07
1.0e+07
6.3e+06
4.0e+06

Figure S9. Contour plots of the same crowded region of a 2D ^{15}N-^{1}H HSQC spectrum of K-Ras plotted with different choices of the lowest contour level. The cross-peaks picked by DEEP Picker are indicated according to their amplitudes. Peaks exceeding the LPAC, which was set to 40 times the noise level, are indicated by circles that are color-coded according to their amplitudes (see sidebar). Peaks with amplitudes below the LPAC are indicated by blue crosses. Contour lines are drawn on a logarithmic scale with the lowest contour level set for the three panels at (A) 4, (B) 15, and (C) 40 times the noise level. The figure shows that the cross-peaks indicated by blue crosses, such as those indicated in Panel C, appear to be false positives when, in fact, they correspond to spectral features that are in all likelihood true cross-peaks as can be seen in Panel A. Examples of such cross-peaks include the standalone cross-peak at position (7.83 ppm (^{1}H), 124.5 ppm (^{15}N)) and the shoulder peak at position (7.7 ppm (^{1}H), 122.5 ppm (^{15}N)). These low amplitude cross-peaks are likely to stem from impurities, chemically modified, or aggregated proteins. Since the vast majority of biomolecular NMR studies focuses on major cross-peaks only, such weak cross-peaks remain unassigned and are ignored for subsequent analysis, for example, by filtering them out based on the LPAC cutoff. Nonetheless, as judged by visual inspection, these weak cross-peaks appear to be true peaks that are correctly identified by DEEP Picker. Finally, the shoulder peak at position (8.59 ppm (^{1}H), 119.7 ppm (^{15}N)) is an example of a major, unassigned peak that is correctly picked by DEEP Picker (see Table S2, 5th column).

References

1. Yuan, C.; Byeon, I. J.; Li, Y.; Tsai, M. D., Structural analysis of phospholipase A2 from functional perspective. 1. Functionally relevant solution structure and roles of the hydrogen-bonding network. *Biochemistry* **1999,** *38*, 2909.

2. Tu, S.; Teng, Y. C.; Yuan, C.; Wu, Y. T.; Chan, M. Y.; Cheng, A. N.; Lin, P. H.; Juan, L. J.; Tsai, M. D., The ARID domain of the H3K4 demethylase RBP2 binds to a DNA CCGCCC motif. *Nat. Struct. Mol. Biol.* **2008,** *15*, 419.

3. Bowles, D. P.; Yuan, C.; Stephany, K. R.; Lavinder, J. J.; Hansen, A. L.; Magliery, T. J., Resonance assignments of wild-type and two cysteine-free variants of the four-helix bundle protein, Rop. *Biomol. NMR Assign.* **2018,** *12*, 345.

4. Yuan, C.; Li, J.; Mahajan, A.; Poi, M. J.; Byeon, I. J.; Tsai, M. D., Solution structure of the human oncogenic protein gankyrin containing seven ankyrin repeats and analysis of its structure--function relationship. *Biochemistry* **2004,** *43*, 12152.

5. Timari, I.; Wang, C.; Hansen, A. L.; Costa Dos Santos, G.; Yoon, S. O.; Bruschweiler-Li, L.; Brüschweiler, R., Real-Time Pure Shift HSQC NMR for Untargeted Metabolomics. *Anal. Chem.* **2019,** *91*, 2304.
