# Supplementary material for: DEEP picker is a deep neural network for accurate deconvolution of complex two-dimensional NMR spectra
Source: Nat Commun. 2021 Sep 1;12:5229. doi: 10.1038/s41467-021-25496-5 (PMC8410766; doi:10.1038/s41467-021-25496-5)
Supplement: Supplementary file 2 — Reporting Summary [file 41467_2021_25496_MOESM2_ESM.pdf]

nature research

Corresponding author(s): Da-Wei Li and Rafael Bruschweiler

Last updated by author(s): Jul 27, 2021

Reporting Summary

Nature Research wishes to improve the reproducibility of the work that we publish. This form provides structure for consistency and transparency in reporting. For further information on Nature Research policies, see our Editorial Policies and the Editorial Policy Checklist.

Statistics

For all statistical analyses, confirm that the following items are present in the figure legend, table legend, main text, or Methods section.

n/a	Confirmed	
☒	☐	The exact sample size (*n*) for each experimental group/condition, given as a discrete number and unit of measurement
☒	☐	A statement on whether measurements were taken from distinct samples or whether the same sample was measured repeatedly
☒	☐	The statistical test(s) used AND whether they are one- or two-sided<br>*Only common tests should be described solely by name; describe more complex techniques in the Methods section.*
☒	☐	A description of all covariates tested
☒	☐	A description of any assumptions or corrections, such as tests of normality and adjustment for multiple comparisons
☒	☐	A full description of the statistical parameters including central tendency (e.g. means) or other basic estimates (e.g. regression coefficient) AND variation (e.g. standard deviation) or associated estimates of uncertainty (e.g. confidence intervals)
☒	☐	For null hypothesis testing, the test statistic (e.g. *F*, *t*, *r*) with confidence intervals, effect sizes, degrees of freedom and *P* value noted<br>*Give P values as exact values whenever suitable.*
☒	☐	For Bayesian analysis, information on the choice of priors and Markov chain Monte Carlo settings
☒	☐	For hierarchical and complex designs, identification of the appropriate level for tests and full reporting of outcomes
☒	☐	Estimates of effect sizes (e.g. Cohen's *d*, Pearson's *r*), indicating how they were calculated

Our web collection on statistics for biologists contains articles on many of the points above.

Software and code

Policy information about availability of computer code

Data collection	Data collection was performed on Bruker AVANCE III NMR spectrometers at CCIC NMR.
Data analysis	The DNN was developed using Tensorflow v1.3.

For manuscripts utilizing custom algorithms or software that are central to the research but not yet described in published literature, software must be made available to editors and reviewers. We strongly encourage code deposition in a community repository (e.g. GitHub). See the Nature Research guidelines for submitting code & software for further information.

Data

Policy information about availability of data

All manuscripts must include a data availability statement. This statement should provide the following information, where applicable:
- Accession codes, unique identifiers, or web links for publicly available datasets
- A list of figures that have associated raw data
- A description of any restrictions on data availability

The spectra used in this work are only used to demonstrate the method's and its software implementation's capabilities. A data availability statement is included in the manuscript.

Field-specific reporting

Life sciences study design

All studies must disclose on these points even when the disclosure is negative.

Sample size	New biomolecular NMR methods are typically demonstrated for 1 or 2 protein systems, which is the gold standard in the field. Here, we demonstrate the method on 7 different proteins and a urine sample.
Data exclusions	None.
Replication	The purpose of this work is to test and demonstrate that DEEP Picker can accurately identify cross-peaks of given NMR spectra from different proteins and metabolomics samples. Each of the hundreds of cross-peaks in each spectrum allows an assessment of the performance of DEEP Picker. We did not explicitly replicate the measurements of 2D NMR spectra, which is consistent with the gold standard in the field, as each spectrum has a large number of cross-peaks each of which provides an independent test of the performance of DEEP Picker. NMR spectra are highly reproducible due to the fully automated electronic nature of data collection with auto-shim, temperature control, etc. Therefore, spectra were only repeated to test the dependence of DEEP Picker performance on sample concentration to vary the amount of noise as shown in Figure S7.
Randomization	Each experimental NMR spectrum was treated identically by DEEP Picker. Therefore, additional randomization was neither necessary nor possible.
Blinding	DEEP Picker was trained on synthetic data and then applied to experimental protein systems. Therefore, DEEP Picker was not biased by the results and blinding is not required

Reporting for specific materials, systems and methods

We require information from authors about some types of materials, experimental systems and methods used in many studies. Here, indicate whether each material, system or method listed is relevant to your study. If you are not sure if a list item applies to your research, read the appropriate section before selecting a response.

Materials & experimental systems

n/a	Involved in the study
☒	☐ Antibodies
☒	☐ Eukaryotic cell lines
☒	☐ Palaeontology and archaeology
☒	☐ Animals and other organisms
☒	☐ Human research participants
☒	☐ Clinical data
☒	☐ Dual use research of concern

Methods

n/a	Involved in the study
☒	☐ ChIP-seq
☒	☐ Flow cytometry
☒	☐ MRI-based neuroimaging
